# Forest mosaics, not savanna corridors, dominated in Southeast Asia during the Last Glacial Maximum

**DOI:** 10.1073/pnas.2311280120

**Published:** 2023-12-26

**Authors:** Rebecca Hamilton, Noel Amano, Corey J. A. Bradshaw, Frédérik Saltré, Robert Patalano, Dan Penny, Janelle Stevenson, Jesse Wolfhagen, Patrick Roberts

**Affiliations:** ^a^isoTROPIC Research Group, Max Planck Institute of Geoanthropology, Jena 07745, Germany; ^b^Department of Archaeology, Max Planck Institute for Geoanthropology, Jena 07745, Germany; ^c^School of Geosciences, Faculty of Science, The University of Sydney, Sydney, NSW 2050, Australia; ^d^Australian Research Council Centre of Excellence for Australian Biodiversity and Heritage, Wollongong, NSW 2522, Australia; ^e^Global Ecology | Partuyarta Ngadluku Wardli Kuu, College of Science and Engineering, Flinders University, Adelaide, SA 5001, Australia; ^f^Biological and Biomedical Sciences, School of Health and Behavioral Sciences, Bryant University, Smithfield, RI 02917; ^g^School of Culture, History and Language, College of Asia and the Pacific, Australian National University, Canberra, ACT 2601, Australia; ^h^Department of Anthropology, College of Liberal Arts, Purdue University, West Lafayette, IN 47907; ^i^School of Archaeology, University of the Philippines, Quezon City 1101, The Philippines

**Keywords:** grassland, palaeoenvironmental change, ecological regime shift, monsoon forest

## Abstract

The dominant paradigm is that large tracts of Southeast Asia’s lowland rainforests were replaced with a “savanna corridor” during the cooler, more seasonal climates of the Last Glacial Maximum (LGM) (23,000 to 19,000 y ago). This interpretation has implications for understanding the resilience of Asia’s tropical forests to projected climate change, implying a vulnerability to “savannization”. A savanna corridor is also an important foundation for archaeological interpretations of how humans moved through and settled insular Southeast Asia and Australia. Yet an up-to-date, multiproxy, and empirical examination of the palaeoecological evidence for this corridor is lacking. We conducted qualitative and statistical analyses of 59 palaeoecological records across Southeast Asia to test the evidence for LGM savannization and clarify the relationships between methods, biogeography, and ecological change in the region from the start of Late Glacial Period (119,000 y ago) to the present. The pollen records typically show montane forest persistence during the LGM, while *δ*^13^C biomarker proxies indicate the expansion of C_4_-rich grasslands. We reconcile this discrepancy by hypothesizing the expansion of montane forest in the uplands and replacement of rainforest with seasonally dry tropical forest in the lowlands. We also find that smooth forest transitions between 34,000 and 2,000 y ago point to the capacity of Southeast Asia’s ecosystems both to resist and recover from climate stressors, suggesting resilience to savannization. Finally, the timing of ecological change observed in our combined datasets indicates an ‘early’ onset of the LGM in Southeast Asia from ~30,000 y ago.

## Significance

We present new qualitative and statistical analyses of 59 palaeoecological records across Southeast Asia to show that, instead of swings between open savanna and dense rainforest ecosystems, the climatic changes of the Last Glacial Period (119–11.7 ka) and particularly the Last Glacial Maximum (conventionally ~23–19 ka) involved fluid transitions between lowland rainforest, more open canopy seasonally dry forest, and tropical montane forest. This “hybrid” open forest biome provides an alternative to the currently accepted binary ecologies for the region and yields new insights into ecological resilience for tropical forests in Southeast Asia and beyond. Additionally, it points to diversified rather than overturned resource availability for humans that were occupying and migrating through the region.

**Fig. 1.**
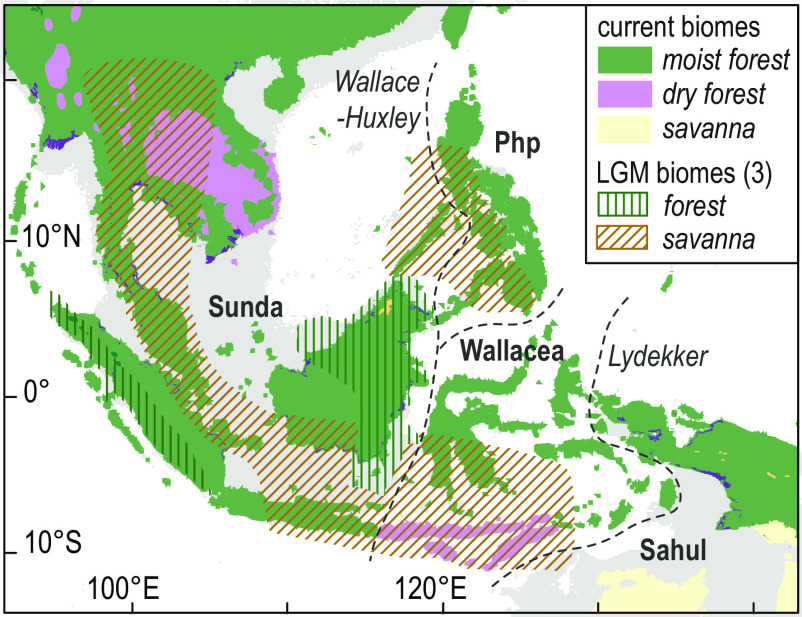
Map of the current biomes of mainland and insular Southeast Asia split into four biogeographic zones: Sunda, Wallacea, Philippines excluding Palawan (Php), and Sahul. These zones are defined by the Wallace–Huxley and Lydekker lines. Dominant biomes are shown relative to the location of the proposed LGM savanna corridor (orange hatch) and forest refugia (green hatch) proposed by ref. 12 and since updated (3, 9). Light gray shading shows the exposed continental shelf at the LGM (~120 m below current sea level) (13).

The presence and extent of a Last Glacial Period (LGP; 119 to 11.7 ka, 1 ka = 1,000 y ago) “savanna corridor” in Southeast Asia is a contested topic in archaeology, palaeoanthropology, and biogeography (1–5). Drier, cooler, and more seasonal climates coinciding with a sea level low stand (~120 m lower than today) during the Last Glacial Maximum (LGM; conventionally ~23 to 19 ka) (6) have been associated with the expansion of grassland ecosystems across large regions of Sundaland (the landmass then connecting mainland Southeast Asia with Sumatra, Java, and Borneo), Wallacea (Sulawesi), and the Philippines (Fig. 1) (3, 7–10). This savanna corridor might have initiated widescale extinction, migration, and replacement of Pleistocene fauna (i.e., faunal turnover) and provided suitable land connections and habitats for the migration of anatomically modern humans (9, 11) (Fig. 1).

The assumed widespread presence of savannas during the LGM has also been used to highlight the sensitivity of some of the world’s most diverse tropical forests to future ecological changes (3, 12). The implications are important for several reasons. First, extant tropical forests—the overwhelmingly dominant biome in the region today (Fig. 1)—directly provide ecosystem services (e.g., food, fuel, nutrient stock) for 9% of the global population. Second, these ecosystems, most of which are considered global conservation priorities (14), sequester carbon, with ramifications for the Earth’s climate (15). Third, an abrupt and region-wide state shift from tropical forest to savanna (“savannization”) is a process that is conceivable under current climate and land-use trajectories in the well-studied Neotropics (16). Savannization could disrupt patterns of biodiversity (17) and push the Earth system onto a new, self-reinforcing trajectory towards long-term hot and dry climates (18). Trends in savannization across mainland Southeast Asia are less studied but appear to be increasing due to fire disturbance and high atmospheric water demand (19): Stressors that will only worsen under current land-use trajectories (20–22) and rising temperatures (23). Although the climate and land–ocean configuration of the LGM are not directly analogous to contemporary conditions, understanding whether tropical forests in Southeast Asia abruptly transitioned to savanna during drier (24, 25), potentially more seasonal (4) climates could shed light on the sensitivity of today’s forests to important stressors, including monsoon precipitation extremes (26), fragmentation, and heightened fire activity (21, 22). The timeliness of this work is highlighted in the Intergovernmental Panel on Climate Change Sixth Assessment Report, which lists nearly all of tropical Asia as a “priority place” for studying climate change impacts and singles out lowland tropical forest as a biome that is poorly understood in terms of acclimatization capacity (26).

The LGM savanna corridor model originated from a review of palaeoenvironmental data published in the 1990s (12). The review concluded that the LGM ecosystems of Sundaland, Wallacea, and the Philippines comprised montane forests which, under conditions 2 to 6° C cooler than today, expanded downslope by 200 m to 1,700 m. It was hypothesized that lowland environments extending across the Sunda Shelf and other rain shadows (southern Borneo, Java, and the south-western Philippines) were dominated by seasonal forest and savannas. A 2005 synthesis of this model (9) concluded that open vegetation likely expanded northward from southern Sundaland toward the Equator during the LGM, but that the LGM landscapes of central Sundaland and the Northern Hemisphere (sub)tropics were difficult to interpret due to sparse and conflicting data. Both reviews (9, 12) offer a tempered classification of “open” vegetation that could comprise either seasonally dry tropical forest or savanna. However, recent research more strongly advocates for the broad replacement of forest with a lowland savanna corridor during the LGM, implying the sensitivity of tropical forests to ecological reorganization (7, 10, 27). This vision is typically premised on single-proxy *δ*^13^C studies of bat guano, sedimentary leaf waxes, or tooth enamel that use variable thresholds of *δ*^13^C to infer past habitat types. It is common for interpretations from *δ*^13^C studies to draw habitat binaries between those hosting plants using C_3_-photosynthetic pathways (rainforest) and those using C_4_-photosynthetic pathways (savannas) (7, 10, 27). Importantly, this binary system of classification can overlook the *δ*^13^C signatures of seasonally dry tropical forests that host C_3_ and C_4_ plants (28). Although rare, contemporary isotopic signatures from seasonally dry forest show that *δ*^13^C_plant wax_ centers on ~25‰ (29), generally overlapping with the savanna classification (7, 25, 27).

**Fig. 2.**
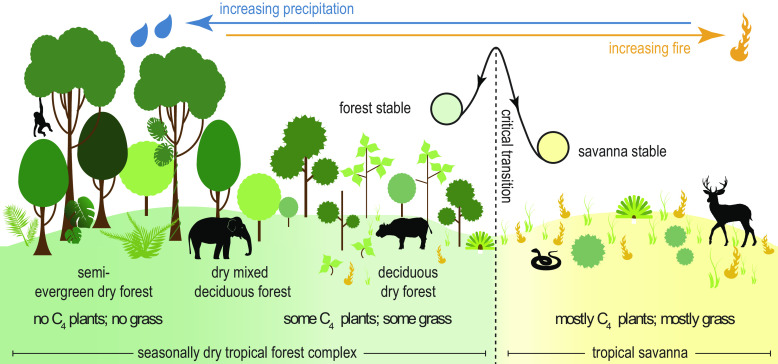
Conceptual diagram showing gradual, smooth transitions between closed and open forest units within the seasonally dry tropical forest complex, and abrupt, potentially difficult-to-reverse transitions between forest and savanna (32). This model illustrates the importance of understanding the past transitions of Southeast Asian forests to predict the resilience of these systems to future precipitation and fire drivers.

Ecological definitions of savanna vs. seasonally dry tropical forest in Asia are complex and debated (30). On the one hand, more open canopy units (termed “dry deciduous forest”) (31) within the naturally mosaicked complex of seasonally dry tropical forest could be classified as savanna given that they host fire-tolerant, mixed tree–C_4_ grass systems (e.g. ref. 30). The other argument (e.g., ref. 32) considers tropical savanna an alternative stable state to tropical forest, and that transitions between forest and savanna resulting in abrupt losses of tree cover are positively reinforced by climate and fire, and are difficult to reverse (33, 34). Because Asia’s seasonally dry tropical forest complex smoothly and reversibly transitions from closed, fire-exclusionary forest units (semi-evergreen dry forest) (31) to dry deciduous forest in response to fire and monsoon precipitation stressors, it functionally behaves as a forest (32) (Fig. 2). Rather than representing an alternative stable state, Southeast Asia’s seasonally dry forests might contribute to the resilience of lowland tropical forest by buffering conditions like heightened seasonality or fire that could otherwise trigger an abrupt and difficult-to-reverse state shift to a true, open savanna (33, 34) (see Fig. 2 for a conceptual diagram of this process). Understanding past smooth transitions between rainforest and seasonally dry forest, and abrupt transitions between forest and savanna, is important for forecasting the likelihood of future ecological change across tropical Asia, particularly because they are thought to be sensitive to change (35).

Savannization has also played an important role in discussions of hominin migration and adaptation in Southeast Asia (36). Focusing on the appearance of our own species in the region in the Late Pleistocene, some have argued that human populations would have been able to move more quickly through mainland and insular Southeast Asia during periods of savanna expansion (9). In addition, there have been broader claims in archaeological contexts about the challenges faced by humans adapting to tropical forests (37) and the expected importance of C_4_ savannas and the large game species they support to hominin subsistence (38). Nevertheless, growing evidence for varied and specialized adaptations to tropical forests in South and Southeast Asia during the Pleistocene (e.g., refs. 39 and 40) makes understanding the phenomenon of savannization important from the standpoint of human evolution and ecology. Expanding archaeological and genetic records from insular Southeast Asia, starting with migrations from Sunda to Sahul at 65 ka (41), moving into regional settlement from around 46 ka (42, 43) (although potentially as early as 73 ka) (44) and postglacial population dispersals and the growth of exchange networks (45), are still poorly understood regarding environmental and habitat change. For example, it has been argued that LGM drying and forest contraction in Southeast Asia gave way to wetter conditions and rapid forest expansion in the terminal Pleistocene, which encouraged hunting shifts towards arboreal resources (46). However, this idea is still debated given that some data suggest the stability of forest cover through the LGM (e.g., ref. 47, but also see the variability in the interpretation of LGM ecological change outlined in *SI Appendix*, Table S1).

Here, we compile and analyze palaeoecological data from across mainland and island Southeast Asia to test explicitly whether a widespread savanna corridor expanded across the region during the LGP and particularly during Marine Isotope Stage 2 (MIS 2) (29 ka to 12 ka). Specifically, we classified habitat types from 59 palaeoecological records to describe the nature and distribution of regional vegetation within different marine isotope stages spanning the LGP and Holocene. Second, we use unique statistical tools to harmonize 26 time-transgressive records that are proxies for habitat openness leading into and out of MIS 2 (34 to 2 ka) to assess: i) the extent to which the canopy opened across the region during MIS 2, indicating the degree to which closed forest was replaced by either savanna or seasonal forest; ii) if and how patterns of opening link to different methodological or biogeographic factors, permitting an exploration of both the different sensitivities of proxies used to gauge habitat change as well as biogeographic features that might influence ecological resilience; and iii) the timing of ecological transitions, refining our understanding of the onset of the LGM in tropical Asia. Collectively, we aim to advance knowledge about the past acclimation capacities of tropical ecosystems in Asia during important phases of climate change and human migration and settlement.

## Results

**Fig. 3.**
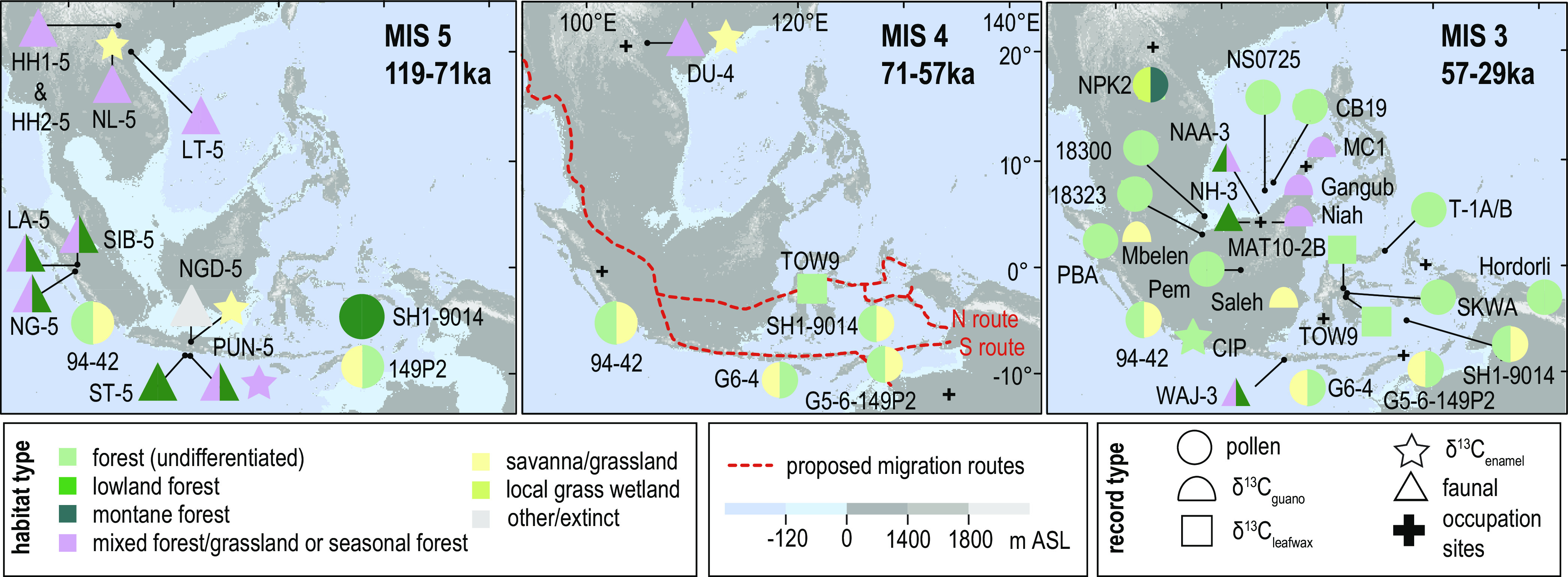
Maps showing the distribution and summary habitat types for datasets spanning MIS 5d-a to MIS 3. Pollen and faunal habitat classification indicate only the dominant (>33%) habitat types within the selected marine isotope stage (see *SI Appendix, Fig. S1.* for detailed proportional data for habitat types, and the Supplementary Dataset for full site names and relevant publications) (49). Habitat classification of *δ*^13^C_enamel_ follows (11). *δ*^13^C_plant wax_ and *δ*^13^C_guano_ are classified as mixed if mean values fall within the range 25 ± 1‰ following Dubois et al. (29), with savanna or forest classifications made where values were below or above this range, respectively. The proposed potential pathways for human migration across Sunda into Sahul (50) are shown by the dotted red line on the MIS 4 time slice.

We classified average marine isotope stage habitat types based on 23 pollen, six *δ*^13^C_guano_, seven *δ*^13^C_plant wax_, 16 faunal fossil, and seven faunal *δ*^13^C_enamel_ sites spanning 119 ka to 0 ka. We also extracted and stratigraphically plotted proxies for vegetation openness drawn from pollen, *δ*^13^C_guano_, and *δ*^13^C_plant wax_ data. The spatial and sampling distribution of these records are presented in *SI Appendix, Supplementary Text 2*. There are few datasets available from the region for MIS 5d–a (119 to 71 ka) and MIS 4 (71 to 57 ka) (Fig. 3), making interpretations of habitat for this period tentative (Fig. 3). Nonetheless, available data for MIS 5, skewed for the zones 10° S to ~0° and >20° N, suggest the presence of closed forest in the equatorial zone and “mixed vegetation” (a general classification including grassland, seasonal forest, and secondary forest) across the region more broadly (Fig. 3). MIS 5d-a habitat types appear to persist or slightly open in MIS 4 (Fig. 3). However, MIS 4 datasets are predominantly palynology records from the southern margin of the study region that capture pollen transported from the savanna ecosystems of mainland north Australia (48) (see *SI Appendix*, Table S1 for original interpretations of pollen source area). Including an Australian grassland signal within the pollen record likely overemphasizes interpretations of canopy openness for southern Southeast Asia. Records from MIS 3, which are more methodologically and spatially diverse, indicate that closed forest dominated the region, with some mixed forest elements within the southern part of the study area (Fig. 3). Although there are fewer faunal data available from the northern part of the study area than for MIS 5d to MIS 4, pollen data from central Thailand indicate the regional presence of montane forest during MIS 3.

**Fig. 4.**
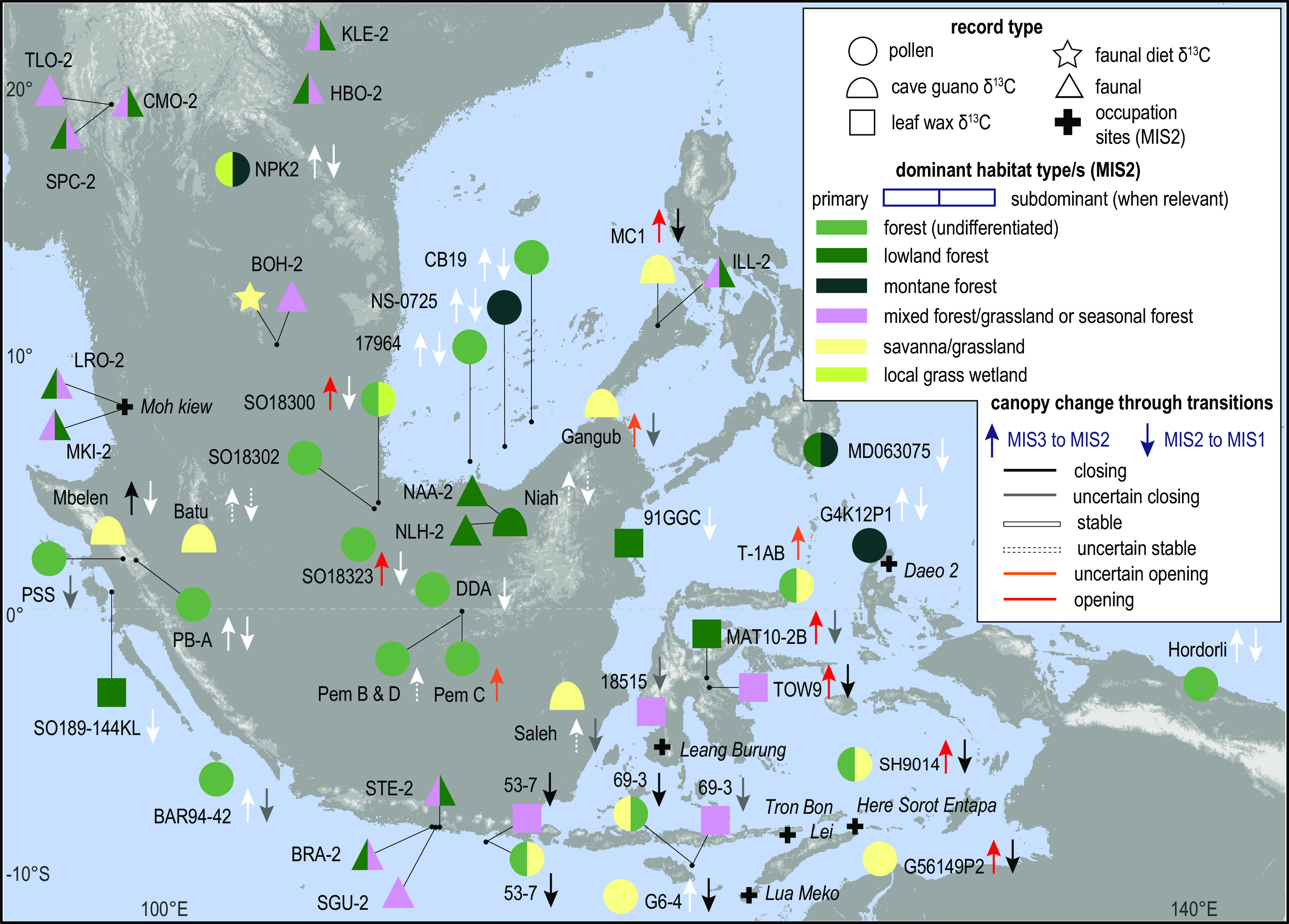
Map summarizing the dominant MIS 2 habitat types and evidence for forest opening. Habitat-type summaries follow those described in the caption for Fig. 3. Arrows showing assessed changes in “openness” from time series datasets (MIS 3 to MIS 2 = up arrow; MIS 2 to MIS 1 = down arrow) are allocated an “uncertain” classification where the record was not resampled and quantitatively analyzed due to a lack of chronological data. We assigned “stable” values where resampled, time-buffered curves did not inflect between marine isotope stages (*SI Appendix*, Figs. S2 and S3).

MIS 2 broadly supported closed forest within equatorial Sunda, with more seasonal forest, savanna, and mixed forest/savanna signals from what is today’s mainland Sunda, Wallacea, and along the southern margin of the study area (Fig. 4). Reconstructed canopy cover change between MIS 3 and MIS 2, and between MIS 2 and MIS 1, show that records from the eastern margin of Sunda and Wallacea were slightly more susceptible to opening during MIS 2 (Fig. 4). However, a pattern that is much more apparent from the data is that the *δ*^13^C_plant wax_, *δ*^13^C_enamel_, and especially the δ^13^C_guano_ records consistently indicate the presence of more open habitat types in MIS 2 than the pollen records show (Fig. 4). Habitats inferred from the *δ*^13^C records include mixed forest/grassland or seasonal forests (when MIS 2 *δ*^13^C averaged −25 ± 1‰) (29), savanna (when average *δ*^13^C > −25 ± 1‰) or, in the Sunda equatorial zone (~3° S – 3° N), forest.

**Fig. 5.**
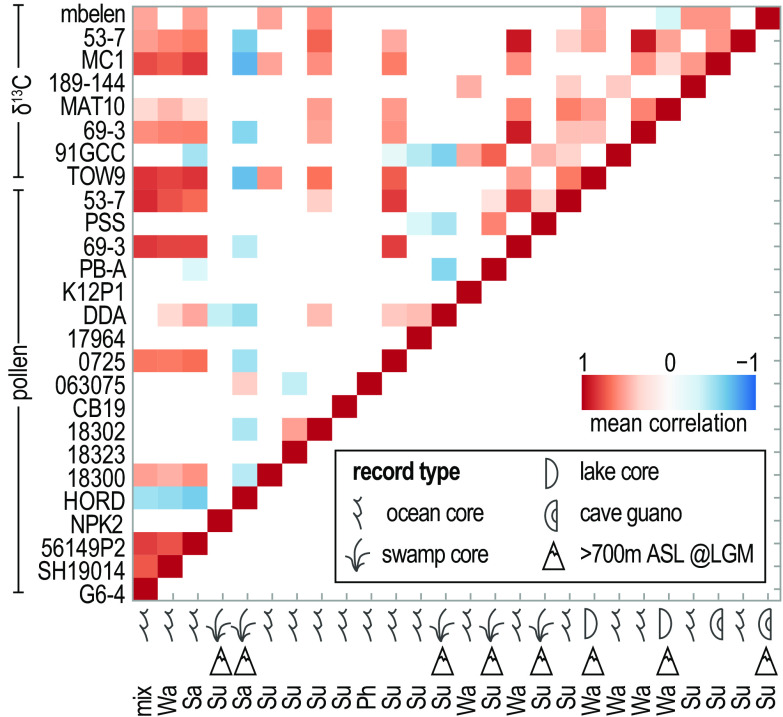
Weighted Pearson correlation matrix showing the relationship between openness proxies developed from pollen and *δ*^13^C records that met the criteria for comparative analysis. Blank spaces reflect no evidence for a correlation with 95% confidence. Mix = mixed signal; Su = Sunda, Wa = Wallacea; Sa = Sahul as defined in Fig. 1.

Correlation of the canopy openness proxies produced for most of the time-transgressive datasets (*n* = 26) (shown as individual time series in *SI Appendix, Supplementary Text 2*) establishes biogeographic or methodological links to vegetation opening in MIS 2. Our analysis reveals clear interrelationships among proxy type, site type, and site elevation between late-stage MIS 3 (34 ka) and 2 ka. There are typically stronger positive correlations between *δ*^13^C records than there are for the pollen proxies of openness (Fig. 5). Additionally, records from lowland sites (<700 m above LGM sea level) (15) that are almost always oceanic (today) or cave records tend to be either positively correlated or uncorrelated. Most upland records (>700 m above LGM sea level) derived from smaller catchment swamps/lakes and caves are either negatively correlated or do not correlate (Fig. 5).

**Fig. 6.**
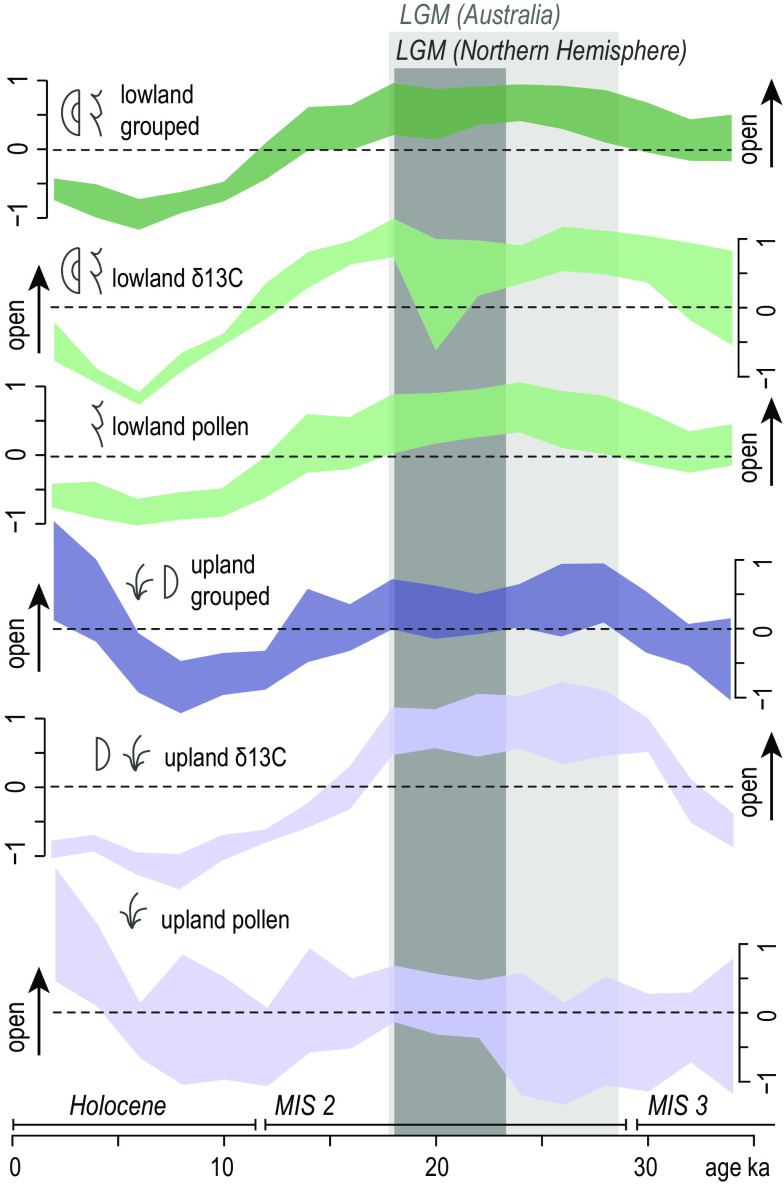
Combined summary plots of scaled openness proxies that met the criteria for comparative analysis. These are grouped into categories established from the correlation analysis, i.e., proxy type (pollen vs. *δ*^13^C) and elevation/basin size (large, lowland basins <700 m above LGM sea level vs. small, upland basins >700 m above LGM sea level). Data are plotted within the age window selected for comparative analysis (34 ka to 2 ka) and shown relative to Northern Hemisphere-biased boundaries conventionally used to define the LGM (23 ka to 19 ka) (51) and those more recently proposed for Australia (28.6 ± 2.8 ka to 17.7 ± 2.2 ka) (52).

We used the two categories established from the correlation analysis—i.e., i) proxy type (pollen vs. δ^13^C records) and ii) elevation-site type (lowland, large-basin sites vs. upland, small-basin sites)—to combine datasets, assess the volatility of these groups, and define major points of change (Fig. 6). Our results indicate that lowland ecosystems closed between 18 ka–14 ka and 10 ka–6 ka. The study region’s lowlands were also slightly more open between 32 ka and 20 ka–18 ka, although this pattern only emerged clearly when we isolated the *δ*^13^C records. Our assessment of the opening of the uplands depends on which proxy we used to gauge habitat openness. Pollen data indicate a stable flora between >34 ka and 6 ka, but *δ*^13^C data show canopy opening between 34 ka and 30 ka, and subsequent closing from 18 ka to 8 ka.

## Discussion

Our results on the extent to which forest canopies opened across the region during the LGP and specifically MIS 2, broadly indicate that closed forest was able to persist, at least in the form of mosaics, across mainland and insular Southeast Asia within the proposed LGM savanna corridor zone (delimited on Fig. 1). However, more open habitat types, including mixed vegetation, seasonally dry tropical forest, and savannas might have expanded in the region during MIS 4 and MIS 2 (Figs. 3 and 4). Our analyses of openness proxies between 34 ka and 2 ka, which capture major transitions between MIS 3 and the Holocene, provide important insights into regional climatic-ecological dynamics. The clear discrepancy in ecological responses between highland and lowland sites suggests the presence of two main habitat types across the region during MIS 2 (Fig. 6). The uplands supported climatically resistant montane forest that remained relatively unchanged from their MIS 3 (and perhaps earlier) state. More dynamic “mixed” ecosystems expanded in the lowlands between ~32 ka and 14 ka–12 ka. These could represent either mosaicked seasonally dry tropical forest similar to spatially restricted monsoonal forest ecoregions within mainland Southeast Asia and Southeast Indonesia today (Fig. 1) (5), or the presence of patchy riparian forests from river systems draining the exposed Sunda shelf. The consistency of the mixed forest signal from lowland records that are distal from the Sunda’s river systems, including non-riparian Sunda shelf records (e.g., SO18323, SO18302) (53), and the 13 then-oceanic and non-Sunda shelf sites (Fig. 4) that capture a large pollen source area, lends weight to the interpretation of regionally representative seasonally dry forest rather than local-scale riparian forest set within an open savanna. The broad-scale presence of forest generally aligns with original interpretations of MIS 2 vegetation from pollen data (*SI Appendix*, Table S1). Overall, our results imply smooth transitions from more closed to more open seasonal forest types during drier, more seasonal climates, rather than an abrupt reorganization of forest into a uniform, open, savanna corridor.

With few exceptions (5, 54), seasonally dry tropical forest has been largely overlooked as a dominant MIS 2 habitat within recent palaeoecological research from Southeast Asia, which we attribute to methodological biases in interpretations of landscape opening. Specifically, pollen and *δ*^13^C studies are skewed toward interpretations of closed forest and savanna, respectively, solidifying the forest-savanna dichotomy (e.g., refs. 7, 10, 27, and 55). Fingerprinting seasonally dry tropical forest within preexisting lowland pollen records is elusive because, unlike montane forests where several dominant taxa are wind-pollinated (e.g., Fagaceae and tropical conifers), pollen production and dispersal within predominantly insect-pollinated, seasonally dry tropical forest tree dominants is limited (56). In some cases, this could lead to local lowland forest pollen signatures being overwhelmed by montane forest signals, particularly because all lowland sites we analyzed from MIS 2 had large catchment areas and are therefore already biased toward capturing the regional vegetation (24). Furthermore, without detailed taxonomic work (e.g., ref. 56), splitting pollen from seasonally dry and ever-wet lowland forest dominants, often from the same family (e.g., Dipterocarpaceae), can be challenging and, with few exceptions (29, 54) closed and more-open lowland forest types tend to be grouped.

*δ*^13^C increases in lowland MIS 2 records are commonly interpreted to reflect the widespread expansion of C_4_ grasses in the landscape and provide evidence for the LGM savanna corridor model (3, 7, 10). However, this overlooks that seasonally dry tropical forests host C_4_ grasses in the understorey (28) and produce more enriched isotopic signatures than their ever-wet counterparts (29). Rather than reflecting an abrupt transition between ever-wet forest and savanna, higher *δ*^13^C in the combined lowland and upland records within the range of seasonal forest (*SI Appendix*, Fig. S2), in combination with the lack of widespread grassland signatures evident in regional pollen records, probably reflects expansion of seasonally dry forest in the lowlands and, potentially, the encroachment of some seasonal elements into the montane zone (Fig. 6). This interpretation aligns with shorter-term ecological (57, 58) and late-MIS 2 and Holocene palaeoecological observations (32, 54) demonstrating the role of seasonally dry tropical forest in supporting forest recovery and resistance to drier climates and fire. Our interpretation of a MIS 2 seasonal forest corridor in the lowlands reflects the original, more tempered classifications of savanna corridor vegetation (9, 12) and underscores the importance of incorporating mixed vegetation types into analyses of palaeoenvironmental *δ*^13^C in Asia and the global tropics.

Our results refine the timing of environmental transitions associated with the LGM in Southeast Asia, with a clear expansion of a more open flora in the lowlands between 30 ka and 12 ka. This “early” and protracted LGM relative to that defined for the Northern Hemisphere (~23 ka to 19 ka) (6) aligns with palaeoenvironmental evidence from parts of South Asia, island Near Oceania, and Australia (52, 59, 60). The timing of this transition coupled with our insight into ecological responses to MIS 4 and especially MIS 2 climates provides new perspectives on how people used and adapted to landscape change. Rather than the wholescale replacement of lowland forest with expansive savanna, the downslope migration of montane forest and increase in mosaicked seasonal forest with grass elements in the lowlands sustained, and likely diversified, resource availability. This perspective, however, comes with the caveat that our data lack the temporal resolution to assess punctuated ecological change associated with shorter-term MIS 2 climate events in the region (e.g., Heinrich Event 1 or the Younger Dryas) that might have influenced subregional-scale resource availability. Nonetheless, the stability or expansion of resources across the LGP is supported by a lack of major faunal turnover during that period (61) in contrast to the early and middle Pleistocene (11). Evidence for human occupation and use of tropical forest resources across Southeast Asia supports the growing evidence for diverse, specialized human adaptations to tropical forests elsewhere in Asia (46, 62, 63). Seasonally dry forest mosaics might have also interacted with fire technology and early anthropogenic burning to maintain vegetation diversity as has been argued for the Niah Caves in Borneo at ~45 ka (64) and more recently for contemporary tracts of seasonally dry tropical forest in mainland Southeast Asia (32).

From an ecological perspective, the continued presence of forest (or mosaicked forest) habitats throughout the LGP, particularly in the cooler, drier, and more seasonal climates of MIS 2, presents a counterpoint to the perception of Asian tropical forests as vulnerable to major change (3, 35). Our analysis of the MIS 2 landscape indicates that Southeast Asia’s forests exhibit two aspects of ecological resilience to climate change: resistance and recovery (65). The persistence and likely range expansion of montane forest through MIS 3 into MIS 2 indicate the capacity of this forest type to resist (and thrive in) more water-limited and seasonal climates. On the other hand, the tendency of lowland forest to transition smoothly between ever-wet and seasonally dry types indicates the elasticity of these systems and their capacity to recover from climatic and associated ecological (e.g., fire and grazing) stressors. These smooth transitions highlight that in Asia, seasonally dry tropical forest might buffer lowland forest resilience in response to drier, more seasonal conditions and associated disturbances like fire. At a regional scale, the ways in which Southeast Asia’s forests resisted and recovered from stressors in the past shed light on how tropical vegetation might respond to future climate change, a research gap explicitly identified for the region (26). We highlight two subhabitats—seasonally dry tropical forest and montane forest—that appear important for maintaining ecological resilience. These results suggest that processes of savannization, currently described for the Neotropics (16), might depend on biogeographic factors that vary among tropical ecorealms. Methodologically, we present original techniques to test abrupt vs. smooth ecological transitions across space-time. These methods have the potential to test similar hypotheses of past savannization or woody plant encroachment in response to human and/or climate drivers elsewhere.

## Materials and Methods

### Regional and Temporal Delimitation and Palaeoecological Data Collation.

We constrained our study to tropical Southeast Asia and what is now west insular Sahul. This comprehensively covers the region that is proposed to have supported a savanna corridor during the LGM (3, 7, 9) and includes biogeographically distinct areas: Sundaland (i.e., the extension of Southeast Asia’s continental shelf that was exposed during the LGM), Wallacea, insular Sahul (including Palawan), and the Philippines (Fig. 1). We examined two timeframes. We first collated data from the LGP (119 ka to 11.7 ka) and the Holocene (11.7 ka to 0 ka) to describe broad ecological change from this period, including the time at which people first migrated through and settled in Wallacea and Sahul (see Fig. 3 and (49) for relevant archaeological records). We then selected a timeframe spanning the end of MIS 3 (34 ka) to the late Holocene (2 ka) for statistical analysis to examine evidence for the LGM savanna corridor.

We mined palaeoecological data that provide information on past habitat changes from our study region and timeframes from online palaeo-environmental databases (Pangea, NOAA), inventories (International Union for Conservation of Nature’s [IUCN] Red List of Threatened Species), and published data. This resulted in three distinct data types: 1) palynological records (i.e., pollen and plant spore datasets), 2) vegetation isotope records (*δ*^13^C_plant wax_ and *δ*^13^C_guano_), and 3) faunal point data (skeletal fossils and *δ*^13^C_enamel_). We refined the collated time-transgressive records (i.e., the palynological and vegetation isotope records; *n* = 76) by rejecting those that were in an incorrect format for data extraction, did not have an age-depth profile, were discontinuous with few/no samples from MIS 2 (29 ka to 11.7 ka) (66), recorded a marine or wetland signal that overwhelmed the broader terrestrial signal (discussed in the original interpretations), or replicated a preexisting, better-resolved record. This resulted in 24 pollen and 13 *δ*^13^C records (*n* = 37). We included faunal point data if they could be placed into distinct marine isotope stages (66).

### Classification of Data into Terrestrial Habitat Type and Single-Variable Landscape Openness Proxy.

We analyzed the selected datasets in two ways to assess vegetation change through time. We first classified dominant habitat types for each marine isotope stage (MIS 5d–a, MIS 4, MIS 3, MIS 2, and MIS 1) (66). This was necessary to assess what biomes occurred in the region prior to MIS 2 when data are insufficiently spatially and temporally resolved for quantitative analyses. Second, we selected a single variable from each time-transgressive palynological and isotope record to examine changes in vegetation openness through time. This allowed us to assess any landscape opening during MIS 2 and examine the drivers of these shifts.

We based our habitat classification for the *δ*^13^C records (from plant wax, guano, and tooth enamel) on a review of preexisting literature to establish contemporary ranges for closed tropical forest, seasonally dry tropical forest, and grassland/savanna ecosystems in the region. Biome-scale classification for pollen data was, in most cases, already made by the authors of each dataset. Where relevant, we recalculated the pollen data to reflect dryland pollen percentages against age (i.e., we removed obvious wetland taxa that reflect hyper-local conditions). We compared our harmonized habitat classifications with the original interpretations to ensure that they were structurally comparable (*SI Appendix*, Table S1), and we provide the details of pollen and *δ*^13^C data extraction and habitat classification record-by-record in *SI Appendix, Supplementary Text 3*. In most cases, the chronological models for these datasets needed updating to obtain the median as well as minimum and maximum ages at 95% confidence for the quantitative analyses described below. This permits the assessment of vegetation change that better accounts for imprecise age-depth relationships that are inevitable for pollen and *δ*^13^C time series data. We used Bacon 2.5.0 (67) in R (68) for chronological remodeling, with settings and output plots for each record outlined in *SI Appendix, Supplementary Text 3*.

We based habitat classification for the faunal fossil data on the IUCN Red List of Threatened Species scheme (Version 3.1) for modern animal taxa. We merged several classifications to scale up to biomes, resulting in six categories: 1) montane forest = 1.9 subtropical/tropical moist montane, 2) lowland forest = 1.6 subtropical/tropical moist lowland + 1.7 subtropical/tropical mangrove vegetation above high tide + 1.8 subtropical/tropical swamp, 3) dry forest = 1.5 subtropical/tropical dry, 4) mixed vegetation = 1 forest + 2 savanna + 3 shrubland + 4 grassland, 5) savanna/grassland = 2 savanna + 3 shrubland + 4 grassland, and 6) extinct and domestic taxa (49). We did not consider artificial and introduced vegetation habitat types.

We averaged habitat values for all datasets across 6 MIS time bins: 1) MIS 5d-a (119 ka–71 ka); 2) MIS 4 (71 ka–57 ka); 3) MIS 3 (57 ka–29 ka); 4) MIS 2 (29 ka–11.7 ka); 5) LGM (as classified from predominantly Northern Hemisphere records; 23 ka–19 ka), and; 6) the Holocene (11.7 ka–present). We mapped these as percentage plots using ArcGIS Pro (69) (*SI Appendix*, Fig. S1).

We did a case-by-case assessment of the clearest proxy for canopy cover provided in original publications/datasets to select a single openness proxy from the multivariable pollen records following Hamilton et al. (70). We described this procedure for each record in *SI Appendix, Supplementary Text 3*. These proxies include grassland, grass, and rarely, nonforest or herb counts as a percentage of the dryland or total pollen count. We used the typically single variable *δ*^13^C_plant wax_ and *δ*^13^C_guano_ records directly to infer changes in vegetation openness assuming that *δ*^13^C enrichment reflects a reduction in forest cover (typically, C_3_ trees) and the expansion of grasslands, which tend to use a C_4_ carbon-fixation pathway (29). In the rare cases where multiple n-alkane homologues were developed for the same record, we selected the variable most sensitive to changes in C_4_ graminoids as the openness proxy (71). We plotted the selected datasets stratigraphically (*SI Appendix*, Figs. S2 and S3).

### Synthesis and Comparative Analysis of Time-Depth Openness Proxies.

We statistically analyzed the continuous openness proxies we extracted from pollen, *δ*^13^C_plant wax_, and *δ*^13^C_guano_ to i) test for patterns of canopy opening between MIS 3 and the Holocene, ii) assess the timeframe in which any “opening” occurred for the LGM in the Southeast Asian tropics, and iii) determine links between these patterns of openness and biogeographical or methodological techniques.

We analyzed the openness proxy datasets comparatively if 1) there was at least one sample from what is classified as the LGM (23 ka to 19 ka) and 2) it included a chronological model providing minimum and maximum age ranges to assess uncertainty (*SI Appendix, Supplementary Text 3*). We designed these criteria to be broad and to include as many records across space as possible, including 18 pollen and 8 *δ*^13^C_plant wax_ and *δ*^13^C_guano_ records (*n* = 26).

We took advantage of the full uncertainty expressed in the age estimates provided for each openness proxy time series. For each proxy and each age estimate at point *t* in time, we resampled 10,000 random Normal deviates using the *rnorm* function in R (68) and the estimates of mean (– *x*_*t*_) and SD (${\hat{s}}_{t}$) of age. However, age uncertainty was expressed as minimum ($x_{\min}$) and maximum ($x_{\max}$) values in all proxies, so we estimated ${\hat{s}}_{t}$ assuming a Student’s *t* distribution, where ${\hat{s}}_{t}=\frac{\left(x_{\text{max}}–\;–{\hat{x}}_{t}\right)+\left(–{\hat{x}}_{t}–x_{\text{min}}\right)}{2\left(1.96\right)}$. Then, for each of the 10,000 resampled time series of length *T* (i.e., each of the *T* age estimates resampled independently following the procedure described above), we standardized the openness proxy values to a set interval of 34 ka to 2 ka in increments of 2 k using the *approx* function in R to account for nonstandardized sampling intervals among time series. The period 34 ka to 2 ka is covered by most records and allows change in landscape openness to be observed across MIS 3 to MIS 2, and MIS 2 to Holocene boundaries (i.e., the hypothesized maximum and minimum age boundaries for the establishment of the LGM savanna corridor). Because each of the 10,000 resampled time series has a slightly different interval between its sequential age estimates, the interpolated proxy openness values also differ slightly in each case. Next, we scaled each of these 10,000 time series per proxy using the *scale* function in R and calculated the upper and lower 95% confidence limits for the proxy value in question at each 2 k-increment time slice between 34 ka and 2 ka with the *quantile* function in R.

With the resampled, scaled time series and the geographic coordinates (longitude/latitude) of their source site, we calculated the correlation among them using a weighted Pearson correlation coefficient provided in the package in R (72) to account for a potential spatial autocorrelation structure among sites. For each time period in a series, we assigned a weight to the correlation coefficient based on the global Moran’s I that penalizes highly spatially correlated data so that the more the data of a given time period are spatially autocorrelated, the less the given time period will contribute to the calculation of the global Pearson correlation among time series. The global Moran’s I is a metric that evaluates whether the spatial pattern of data sites is clustered, dispersed, or random based on feature location and values (73). Where the 95% CI did not overlap zero, we concluded there was evidence for a correlation and so we could average the relevant (correlated) time series.

To generate the combined summary plots of scaled openness proxies that met the criteria for comparative analysis (Fig. 6), we grouped the time series into their relevant categories (i.e., lowland, lowland *δ*^13^C, lowland pollen, upland, upland *δ*^13^C, and upland pollen). For each time series in each group, we took 1,000 random uniform samples between the upper and lower confidence limits of the time-standardized, scaled values, calculated the median, and then calculated the upper and lower confidence limits of these medians across all component time series. We provide all code and data to reproduce the analyses described above at https://doi.org/10.5281/zenodo.8098424 (49).

## Supplementary Material

Appendix 01 (PDF)

## Data Availability

Datasets & code data have been deposited in GitHub (https://doi.org/10.5281/zenodo.8098424) (49). Previously published data were used for this work (The paper reviews and remodels pre-existing, open access pollen and d13C data in new ways. All original sources are cited in the *SI Appendix*. Due to the size restrictions of the paper, it was not possible to cite all of the original papers in the main text).
