# Supplementary material for: Forest mosaics, not savanna corridors, dominated in Southeast Asia during the Last Glacial Maximum
Source: Proc Natl Acad Sci U S A. 2023 Dec 26;121(1):e2311280120. doi: 10.1073/pnas.2311280120 (PMC10769823; doi:10.1073/pnas.2311280120)
Supplement: Supplementary file 1 — Appendix 01 (PDF) [file pnas.2311280120.sapp.pdf]

Supporting Information for
Forest mosaics, not savanna corridors, dominated in Southeast Asia during the Last Glacial Maximum.

Rebecca Hamilton[a,b,c,d*], Noel Amano[b*], Corey J. A. Bradshaw[e,d], Frédérik Saltré[e,d], Robert Patalano[a,f], Dan Penny[c], Janelle Stevenson[d,f], Jesse Wolfhagen[2,8], Patrick Roberts[1,2,9]

[a]isoTROPIC Research Group, Max Planck Institute of Geoanthropology, Jena 07745, Germany

[b]Department of Archaeology, Max Planck Institute of Geoanthropology, Jena 07745, Germany

[c]School of Geosciences, Faculty of Science, The University of Sydney, Camperdown, NSW 2050, Australia

[d]Australian Research Council Centre of Excellence for Australian Biodiversity and Heritage, Wollongong, NSW 2522, Australia

[e]Global Ecology | *Partuyarta Ngadluku Wardli Kuu*, College of Science and Engineering, Flinders University, GPO Box 2100, Adelaide, SA 5001, Australia

[f]Biological and Biomedical Sciences, School of Health and Behavioral Sciences, Bryant University, Smithfield, RI 02917, USA

[g]School of Culture, History and Language, College of Asia and the Pacific, Australian National University, Canberra, ACT 2601, Australia

[h]Department of Anthropology, College of Liberal Arts, Purdue University, West Lafayette, IN 47907, USA

[i]School of Archaeology, University of the Philippines, Quezon City 1101, The Philippines

Corresponding Author Emails: rebecca.hamilton@sydney.edu.au; amano@gea.mpg.de

This PDF file includes:

Supplementary Text 1: Supporting information on vegetation changes during the MIS 2, including a summary of previous interpretations of MIS 2 vegetation from the study region and a comparison of how these align with our synthesis and re-analysis of major habitat types.

Supplementary Text 2: A visualization of the individual records analyzed in this study that show the inputs used to reconstruct habitat types and transitions in canopy openness, and the data inputs for correlation and comparative analyses.

Supplementary Text 3: Methods for extracting and synthesizing time-transgressive pollen and isotopic records.

References

Other supporting materials for this manuscript include the following:

Datasets & code are available from: https://doi.org/10.5281/zenodo.8098424

Supplementary Text 1: Supporting information on vegetation changes during MIS 2, including a summary of previous interpretations of MIS 2 vegetation from the study region and a comparison of how these align with our synthesis and re-analysis of major habitat types.

This supporting documentation, organized into Table S1, summarizes interpretations of MIS 2 vegetation change from prior research done in the study region. This is designed to provide additional details on site type and source area that are relevant to the interpretations we made, and to cross-check the compatibility of our simplified habitat classifications (Fig. 4, main text) with the original vegetation reconstructions. We provide a justification for why we excluded some of the records referred to in Table 1 in our analysis in the Materials & Methods and in the supplementary dataset available at https://doi.org/10.5281/zenodo.8098424

Table S1: A summary of the original publications, proxies, and interpretations related to MIS 2 vegetation change from the study region. We include habitat classifications we developed (see main text Fig. 4) to cross-check new analyses with the original findings.

**Reference**	**Study type/ proxy**	**Location**	**Record**	**Vegetation interpretation from the original study**	**Habitat interpretation from the subject study (Fig. 4, main text)**
Kershaw et al. (2011)	Review of pollen records	Southeast Asia	NA	Downslope expansion of montane forests during the LGM.	NA
Flenley (1996)	Review of pollen records	Sunda-Sahul region	NA	Downslope expansion of lower montane rainforests during the LGM. Wide scale disappearance of upper montane rainforest.	NA
Penny (2001)	Pollen	Sunda (Thailand)	NPK2 (swamp)	From 40 ka to the upper Holocene boundary, the region supported a montane Fagaceae-coniferous forest, analogous to the evergreen montane forest of contemporary southwest China. Seasonally dry broadleaved tropical forest expanded in the region during the Holocene, presumably in response to warming conditions. Swamp conditions mean that record picks up abundant local sedge and grass pollen.	Local grass wetland & montane forest
van der Kaars et al. (2010)	Pollen	Sunda (Indian Ocean:	BAR94-42	MIS 2 was characterized by relatively closed vegetation compared to MIS 3 (52 ka to 43 ka). The MIS 2 vegetation of south-western	Forest

		southwest Sumatra)		Sumatra likely comprised montane forest, with closed-canopy forest becoming regionally dominant from 43 ka.	
Newsome and Flenley (1988)	Pollen	Sunda (Sumatra)	Danau di Atas (DDA)	Forest persistence during the LGM.	Forest
Maloney (1980)	Pollen	Sunda (Sumatra)	Pea Sim Sim (PSS)	Mid-montane types (Coniferae and Fagaceae) dominate between 18.5 and 16.5 ka, although some open types are present.	Forest
Maloney and McCormac (1995)	Pollen	Sunda (Sumatra)	Pea Bullok (PB-A)	Expansion of tropical montane forest during the LGM.	Forest
McCarthy et al. (2022)	$\delta 13C_{guano}$	Sunda (Sumatra)	Mbelen Cave	C_4 vegetation present during the Last Glacial Period, but expanded at 25 ka. This sequence does not cover the Holocene.	Savanna/ grassland.
Niedermeyer et al. (2014a)	$\delta^{13}C$ alkanes (n-C30, n-C32)	Sunda (offshore West Sumatra)	SO189-144KL [189-144KL] (ocean)	C_3 plants (tropical forest trees) dominant throughout the LGM and the Holocene.	Forest
Stuijts (1993) in van der Kaars et al. (2000)	Pollen	Sunda (Java)	?	The LGM is characterized by forest stability with a downslope expansion of lower montane forest types between > 20 ka and 12 ka.	NA
van der Kaars and Dam (1997); van der Kaars and Dam (1995)	Pollen	Sunda (West Java)	DPDR-I; DPDR-II (swamp)	Mid-montane forest migrated downslope during the LGM, suggesting temperatures in the region cooled by 3.6 to 7.2 °C.	NA
van der Kaars et al. (2001)	Pollen	Sunda (West Java)	Rawa Danau (RD-3)	The late MIS 2 portion of the record (16.3 ka and 11.25 ka) is dominated by a local open, herbaceous swamp signal. The dryland vegetation within the caldera was likely dryland forest.	NA
Stuijts (1993) in van der Kaars et al. (2000); Newsome and Flenley (1988)	Pollen	Sunda (West Java)	Situ Bayongbong	Downslope expansion of montane forest between > 20 ka and 12 ka. The vegetation at the Situ Bayongbong site coming out of the LGM (16.5 ka to 12.4 ka) was characterized by coniferous mid-montane forest.	NA

Ruan et al. (2019)	$\delta^{13}C$ alkanes (n-C29, n-C 31, n-C 33)	Sunda (East Java)	GeoB 10053-7 [53-7] (ocean)	More positive leaf wax $\delta^{13}C$ during the LGM indicate an expansion of more drought-tolerant C_4 vegetation. $\delta^{13}C$ n-29 suggest the expansion of montane rainforest taxa during the LGM. $\delta^{13}C$ n-33 and $\delta^{13}C$ n-31 suggest the expansion of more open forest or savanna in the lowlands.	Mixed forest/grassland or seasonal forest.
Ruan et al. (2019)	Pollen	Sunda (East Java)	GeoB 10053-7 [53-7] (ocean)	High grass pollen (40%) during the LGM probably reflects a more open-canopy vegetation type locally in lowland East Java. Due to the blocking effect of the ~ 3000 km-long volcanic arc mountain chains from Sumatra to Java, the authors assumed the contribution of pollen taxa from the exposed Sunda Shelf during the LGM were minor. Australian contributions to the overall pollen counts are relatively low but consistent.	Savanna/grassland & forest
Wang et al. (2009)	Pollen	Sunda Shelf (LGM exposed & connected Sumatra, Java & Borneo)	SO18300; SO18232; SO18302 (ocean)	The exposed Sunda shelf during the LGM was covered with humid, rainforest. A marshy vegetation (sedges, reeds, bamboo) fringed with palms and ferns developed in the valley along the North Sunda River. The forest contribution is dominated by lowland types. Lower montane rainforest groups peak in the early LGM (SO18300). There is some evidence for distal, downslope migration of upper montane forest types. An increase in grass (and sedge) pollen during the LGM is thought to be more closely linked to the expansion of local Poaceae- and Cyperaceae-rich swamplands than the broader expansion of dryland open forest or savanna-type vegetation.	SO18300: Local grass wetland & forest<br>SO18232: Forest<br>SO18302: Forest
Sun et al. (2002); Sun et al. (2000)	Pollen	Sunda Shelf slope	SO17962; SO17964 (17964) (ocean)	LGM dominated by forest pollen with a lower fernland and swampland contribution than for the slope flat cores analyzed in Wang et al. (2009).	SO17962: NA<br>17964: forest
Yang et al. (2021)	Pollen	Sunda Shelf flat	CB19 (ocean)	LGM dominated by forest pollen. During this time, upper montane forest expanded and compressed the living space of lower montane and lowland forests.	Forest
Thilakanayaka et al. (2019)	Pollen & $\delta^{13}C_{org}$	Sunda Shelf	NS-0725 (ocean)	This record is interpreted as capturing a fluctuating signal between Borneo (MIS 1 and MIS 3) and the Sunda shelf (MIS 2). During MIS 2, including the LGM $\delta^{13}C_{org}$ record (which captures a terrestrial and marine signal), suggests that the most prominent vegetation type on the Sunda Shelf during the LGM was C_3 plants. The pollen record suggests that this vegetation comprised lowland forest.	Pollen: Montane forest<br>$\delta^{13}C_{org:NA}$

Dubois et al. (2014)	$\delta^{13}C$-30FA	Sunda (Borneo)	BJ8-03-91GGC [91GGC] (ocean)	C3 vegetation persists during the LGM and Holocene.	Forest
Anshari et al. (2004)	Pollen & charcoal	Sunda (Borneo)	PemB; PemC; PemD (lake)	Montane and sub-montane forest expanded during the LGM, presumably under cooler-than-present temperature. There is biome-scale stability across assessed timeframe.	PemB: Forest<br>PemC: Forest<br>PemD: Forest
Hunt et al. (2012)	Pollen	Sunda (Borneo)	Niah cave	The LGM landscape comprised a disturbed, open forest.	NA
Wurster et al. (2019)	$\delta^{13}C_{guano}$	Sunda (Borneo)	Niah cave	C_3-dominant (closed forest) vegetation persisted through the LGM, with $\delta^{13}C$ values between −24.7 and −26.2‰, aside from a brief increase to −22.9‰ at ∼13.4 ka.	Forest
Wurster et al. (2019)	$\delta^{13}C_{guano}$	Sunda (Borneo)	Saleh cave	Open (C_4) vegetation existed during much the past 40,000 years before present, providing the strongest evidence yet for the hypothesized savanna corridor.	
van der Kaars (1991)	Pollen	Wallacea (Molucca Sea)	G4-K12P1 [K12P1] (ocean)	This record likely captures vegetation changes from Halmahera (Maluku). The LGM (i.e., prior to 16.5 ka) was characterized by mid-montane *Lithocarpus/Castanopsis* types and a sharp decline in fern cover. This suggests downslope migration of montane oak forests. There are no palynological indicators of drier conditions.	Montane forest
Dubois et al. (2014)	$\delta^{13}C$-C30FA	Wallacea (Sumba)	GeoB10069-3 [69-3] (ocean)	C_4 grasslands expanded during the LGM. C_3 vegetation increases between 18 ka and 7–5.5 ka (with a slight reversal during the Bølling-Allerød period). Last 2 k years characterized by increasing C_4 plant abundance.<br><br>The location of core 69-3 in the Savu Basin, which is bordered only by narrow shelves, means that higher C_4 herbs input during the last glacial stages cannot solely be from grasslands colonizing the nearby exposed shelves. Rather, tropical rainforest on the islands are thought to be replaced with C_4 herbs.	Mixed forest/grassland or seasonal forest
Dubois et al. (2014)	pollen	Wallacea (Sumba)	GeoB10069-3 [69-3] (ocean)	Pollen from C_4 herbs (unclear as to classification, presumably grass pollen) comprise 30–50% of the count between 30 ka and 17 ka, and gradually reduce in the Holocene (10 and 30%). Lowland forest taxa, inversely made up 20 to 30% of the pollen count between 30 ka and	Savanna/grassland & forest

				17 ka, and increase to > 30% in the Holocene. Upper and lower montane forest are higher pre-17 ka, while monsoon rainforest taxa and “other herbs” remain constant throughout. Upper and lower montane forest are not included in primary paper (only in the SI).	
Russell et al. (2014)	$\delta^{13}C$-C28	Wallacea (Sulawesi)	TOW10-9B (TOW9) (lake)	Rainforest vegetation present during MIS 3 and the Holocene contracted between ~ 33,000 and 16,000 years before resent and were replaced with more open (unspecified) terrestrial ecosystems.	Mixed forest/grassland or seasonal forest
Wicaksono et al. (2017)	$\delta^{13}C$-C28FA	Wallacea (Sulawesi)	SO18515 (18515) (ocean)	Regional grassland expansion during the LGM.	Mixed forest/grassland or seasonal forest
(Wicaksono et al., 2015)	$\delta^{13}C$-C28 FA	Wallacea (Sulawesi)	IDLE-MAT10-2B [MAT10-2B] (lake)	There is an apparent shift from closed rainforest towards more drought-tolerant ecosystems during the LGM. However, the absolute values of LGM $\delta^{13}C$ are not enriched enough to require incorporation of C_4 grasses into the landscape, and could reflect more open, C_3 ecosystems with increased water stress.	Forest
Hope (2001)	pollen	Wallacea (Sulawesi)	SKW-A; SKW-B (swamp)	The LGM and mid- and early- Holocene portion of this record is highly compressed or missing according to chronological modelling described in the paper. The MIS 2 portion of the record that is included in the age-depth model shows an expansion of montane forest pollen in the late Pleistocene. The period between 30 ka and 17 ka, including the LGM, shows downslope expansion of Fagaceae forest. A slight increase in Poaceae pollen over the same period is attributed to either the formation of extra-local secondary grasslands in forest gaps, or the expansion of grasslands and sedgelands locally around the core site when water levels in the mire reduced.	NA
Dam et al. (2001)	pollen	Wallacea (Sulawesi)	T-1A/B (lake)	The period of the record between 30.8 ka and 12.7 ka is missing or highly compressed. This is attributed to reduced lake levels under a drying climate and, likely, the formation of a shallow wetland around the lake during that time. This means that the vegetation signal from MIS 2 captured in the core sediments is dominated by a local, herbaceous assemblage. Regional vegetation change on either side of the LGM is difficult to gauge due to the hiatus and local signal being recorded by the lake sediments. However, the regional vegetation	Savanna/grassland & forest

				appears dominated by a montane forest at around 32 ka to 30 ka, and by a relatively open/seasonal lowland forest signal after 12.7 ka.	
Hamilton et al. (2019b)	Pollen	Wallacea (Sulawesi)	LL2 (lake)	This record indicates a higher prevalence of seasonal/ monsoon forest types and fire at the end of the LGM (i.e., in the millennia following 17 ka). This would suggest the expansion of a monsoonal/ seasonal forest during the peak of the LGM, although the record does not extend back into this period.	NA
Bird et al. (2007)	$\delta^{13}C_{guano}$	Philippines: Palawan (Sunda)	Makangit Cave (MC1)	LGM δ^{13}C as high as -13·5‰ indicate that a C_4-dominant grassland existed in the area. Guano δ^{13}C of -25‰ to -28‰ in the Holocene suggest that this open vegetation was replaced by C_3-dominated closed tropical forest by the mid-Holocene.	Savanna/grassland
Wurster et al. (2010)	$\delta^{13}C_{guano}$	Philippines: Palawan (Sunda)	Gangub Cave	δ^{13}C indicate Pleistocene forest until ∼ 33.5 ka, after which δ^{13}C enrichment (to between −26 and −18‰) suggest replacement with savanna (C_4) vegetation. Rainforest was again present in the cave area by 13.5 ka.	Savanna/grassland
Wurster et al. (2010)	$\delta^{13}C_{guano}$	Philippines: Palawan (Sunda)	Batu Cave	C_4 grasslands were a large component of regional vegetation from at least 35 ka until 16 ka, remaining > −22.6‰ until the end of the LGM. After the LGM, an initial decline in δ^{13}C occurred at ∼14.7 ka, with an increase in δ^{13}C to −23.3‰ between 13.4 ka and 12.5 ka. C_3 vegetation (forest) is evident after 10.5 ka and persisted until the present.	Savanna/grassland
Bian et al. (2011)	Pollen	Philippines (Davao Gulf)	MD06-3075 (ocean)	LGM is characterized by the expansion of tropical montane forest, suggesting downslope migration under a cooler climate.	Montane forest & forest.
van der Kaars et al. (2000)	Pollen	Mixed Wallacea/ Sunda/ Sahul (Banda Sea)	SH-9014 (ocean)	LGM is characterized by the downslope expansion of lower montane forest and replacement of ferns with grasses.	Savanna/grassland & forest
van der Kaars (1991)	Pollen	Sahul (Ceram Trench)	G5-2-056 (ocean)	This record likely captures vegetation changes from New Guinea. The end of the LGM and millennia following (i.e., 17 ka to 10 ka) is characterized by a higher prevalence of woodland and grassland taxa. This suggests a more open forest in New Guinea during the peak of the LGM, though the record does not extend back into this period.	NA

van der Kaars (1991)	Pollen	Sahul (Timor Trench)	G5-6-149P2 (ocean)	This record likely captures a mixed signal derived from Timor, northern Australia and the Sahul shelf. Pollen data from the LGM suggest the expansion of an open woodland-scrubland vegetation with forested rims along rivers.	Savanna/grassland
van der Kaars (1991)	Pollen	Mixed Sahul/ Sunda (Lombok Ridge)	G6-4 (ocean)	This record likely captures a strong signal from the north-western Australia, with some contribution from maritime southeast Asia. The period between 40 ka and 11.7 ka, including the LGM, is characterized by the expansion of grassland taxa.	Savanna/grassland
Hope and Tulip (1994)	Pollen	Sahul (New Guinea)	Hordorli (swamp)	Montane forest grew continuously around the site with an increase in higher altitude forest between 25 ka and 10.5 ka.	Forest
Walker and Flenley (1979)	Pollen	Sahul (New Guinea)	Sirunki	Expansion of montane forest (*Nothofagus/ Castanopsis/ Phyllocladus*) forest during the LGM.	NA

Supplementary Text 2: A visualization of the individual records we analyzed that show the inputs used to reconstruct habitat types and transitions in canopy openness, and the data inputs for correlation and comparative analyses.

An overview of broad-scale habitat classifications for each Marine Isotope Stage (MIS) we included, as well as the LGM (conventionally classified as ~ 23 ka to 19 ka) (6) are in Fig. S1. These data feed into the simplified habitat classification made on Fig. 3 and Fig. 4 in the main text.

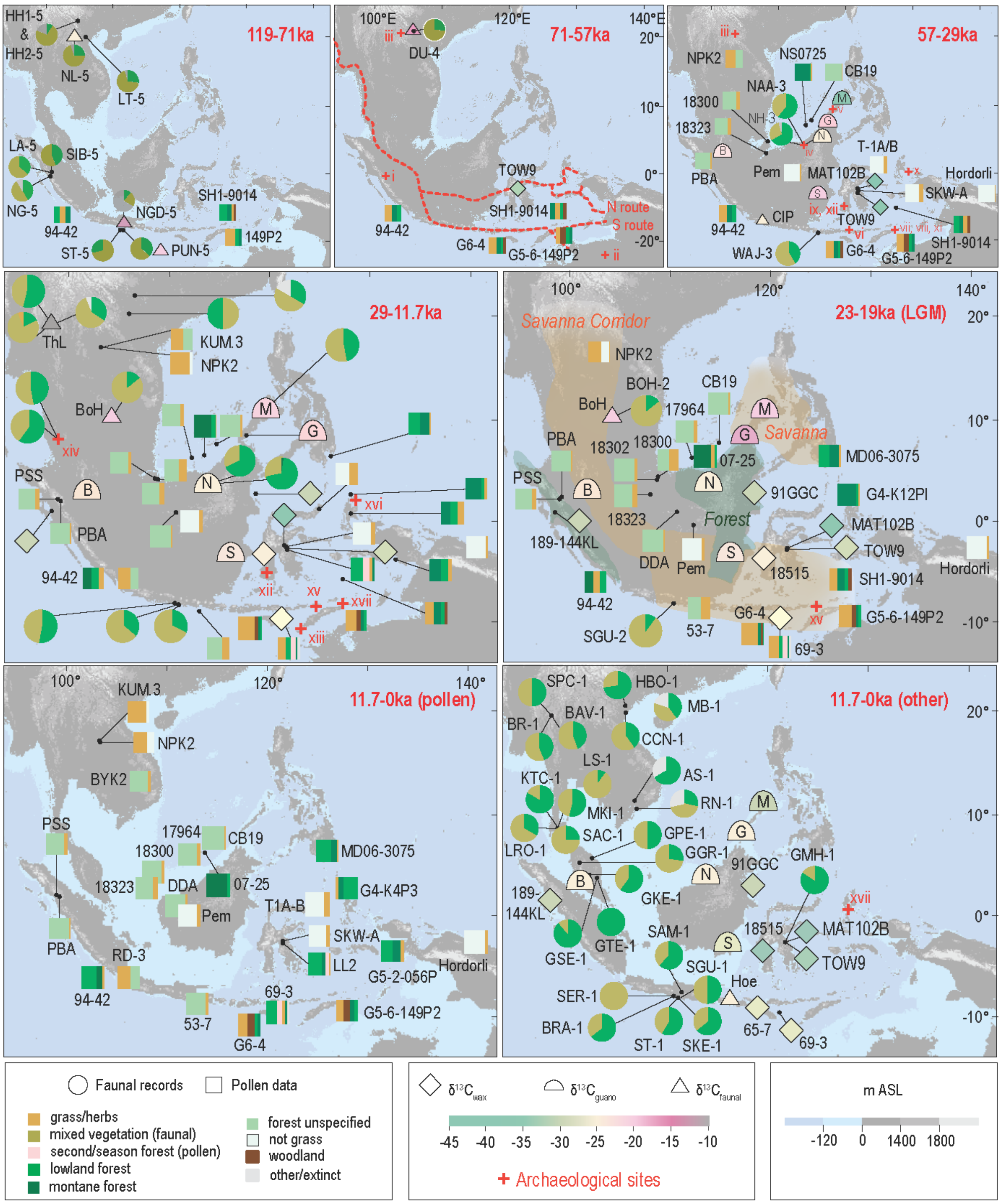

Fig. S1: Maps showing detailed habitat classifications from pollen, isotopic, and faunal proxy data across the time slices we considered (MIS 5 [119 ka–71 ka], MIS 4 [71 ka–57 ka], MIS 3 [57 ka–29 ka], MIS 2 [29 ka–

11.7 ka], the LGM [23 ka–19 ka], and the Holocene [11.7 ka–present]). We averaged data across each selected time bin. Sea-level data drawn from Spratt and Lisiecki (2016), and continental elevation zones are based on contemporary forest unit boundaries (0–700 m = lowland forest; 700–1400 m = hill forest, 1400–1800 m = lower montane forest; ≥ 1800 m = upper montane forest and grassland). Red dashed lines on MIS 4 map (71 ka–57 ka) indicate proposed human migration pathways from Sunda to Sahul by (Birdsell, 1977).

The down-core sampling distribution and age uncertainty windows for the 'canopy openness' time series used for comparative analysis are shown on Fig. S2 (δ^{13}C records) and Fig. S3 (pollen records). For the records that permitted comparative analysis (see Material & Methods in the main text and the supplementary dataset), we used the presence or absence of a curve inflection (grey shading) between MIS 3 and MIS 2, and between MIS 2 and MIS 1, to infer whether there was canopy opening, canopy closing, or no canopy change in response to the onset and termination of the LGM. This is summarized using arrows on Fig. 4 in the main text. The resampled curves (grey shading) serve as data inputs for the correlation and comparative analyses summarized on Fig. 5 and Fig. 6 in the main text.

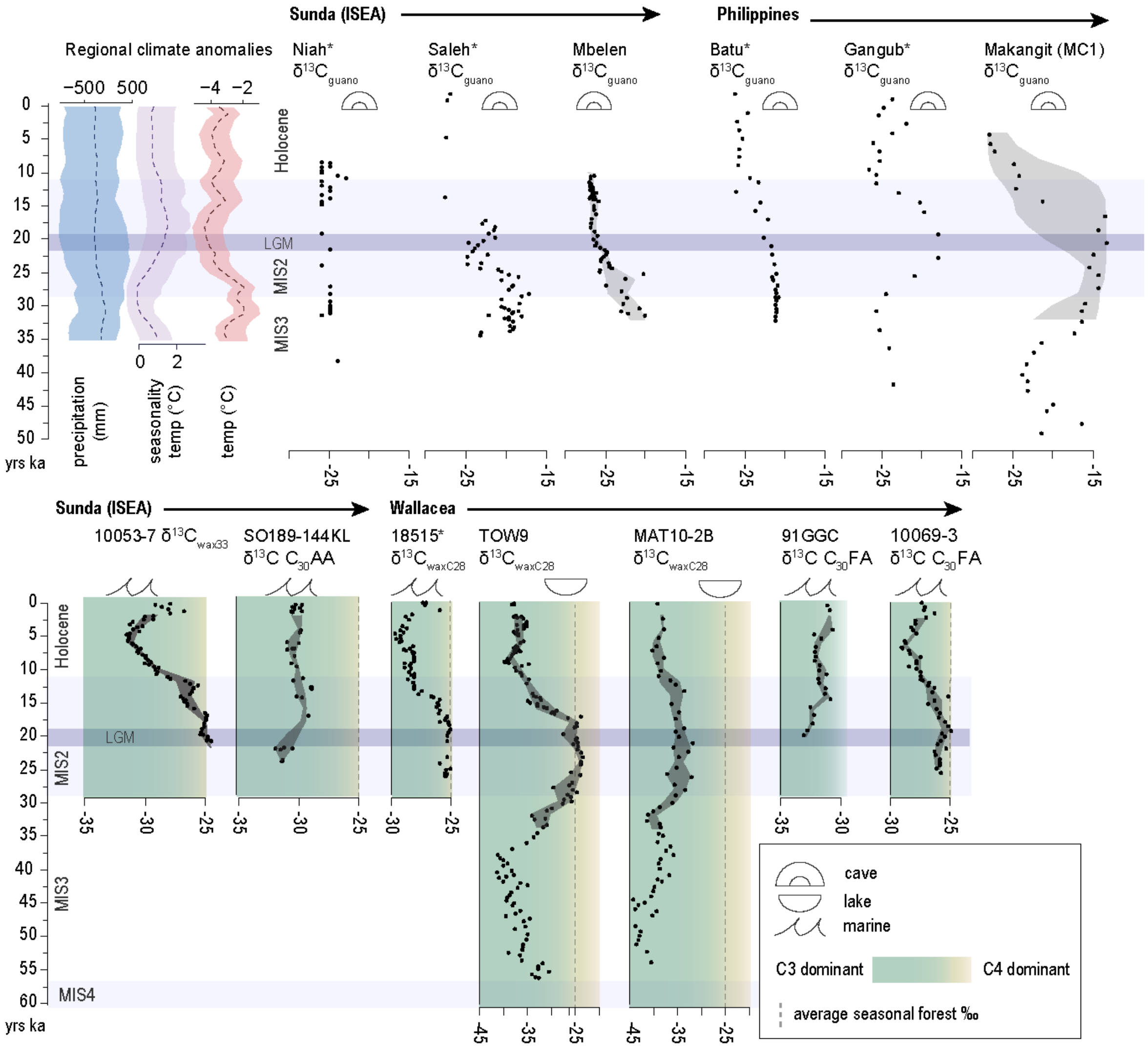

Fig. S2: Stratigraphic plot of δ^{13}C records showing guano (row 1) and sediment plant wax (row 2) records we obtained (black points). Grey shading shows the range boundaries (95% confidence interval) for datasets that we resampled at 2000-year intervals between 2 ka and 34 ka for correlation and volatility analysis, using the minimum and maximum ages calculated from age-depth modelling of the sequences (see Supplementary Text

3). Data plotted against climate anomalies (Hadley CM3) calculated for the region (20º N–11º S; 98º E–141º E) across the same period.

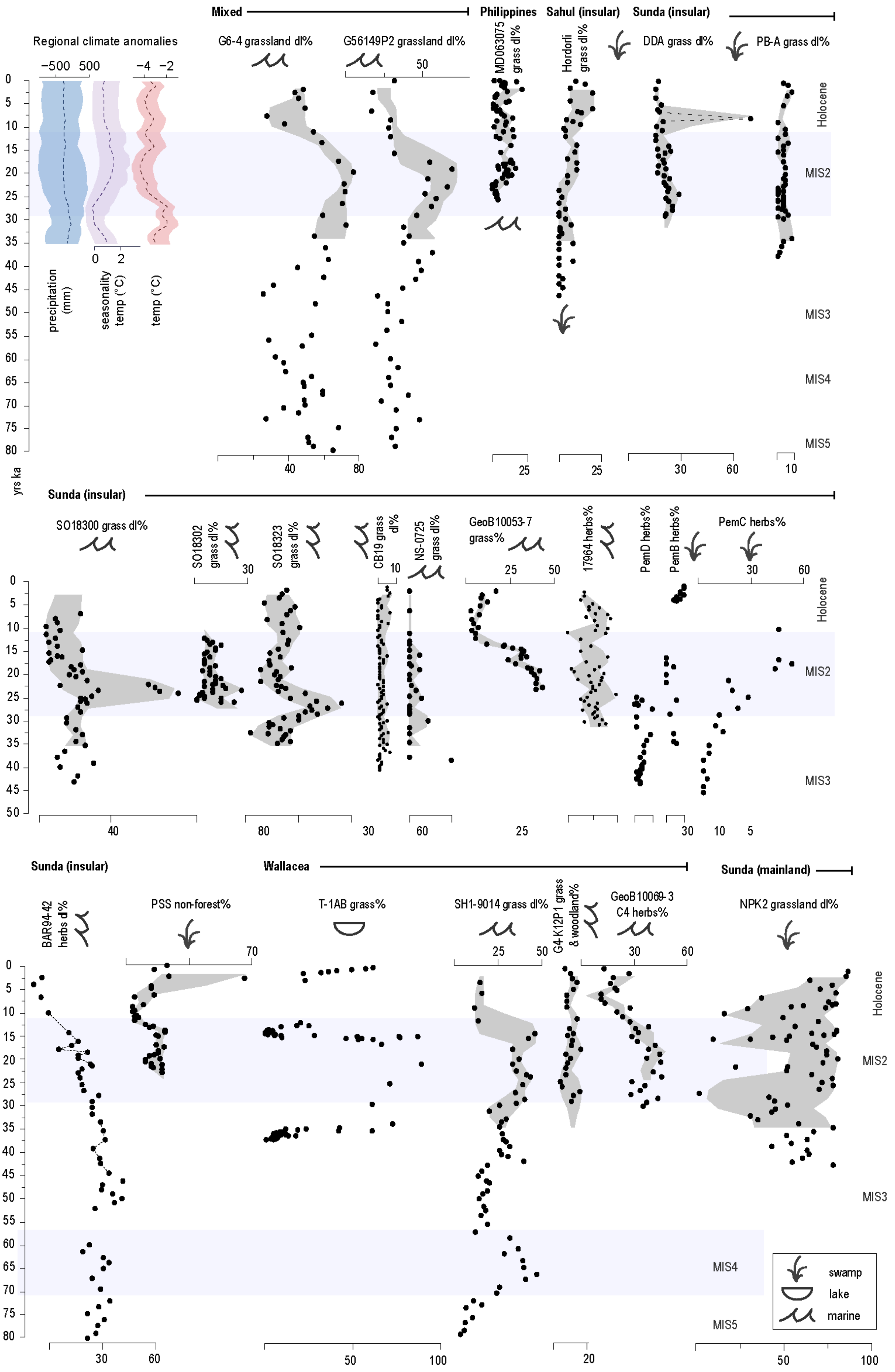
Mixed
Philippines
Sahul (insular)
Sunda (insular)
Regional climate anomalies
G6-4 grassland dl%
G56149P2 grassland dl%
MD063075 grass dl%
Hordorli grass dl%
DDA grass dl%
PB-A grass dl%
precipitation (mm)
seasonality temp (°C)
temp (°C)
yrs ka
Holocene
MIS2
MIS3
MIS4
MIS5
SO18300 grass dl%
SO18302 grass dl%
SO18323 grass dl%
CB19 grass dl%
NS-0725 grass dl%
GeoB10053-7 grass%
17964 herbs%
PemD herbs%
PemB herbs%
PemC herbs%
Wallacea
Sunda (mainland)
BAR94-42 herbs dl%
PSS non-forest%
T-1AB grass%
SH1-9014 grass dl%
G4-K12P1 grass & woodland%
GeoB10069-3 C4 herbs%
NPK2 grassland dl%
swamp
lake
marine

Fig. S3: Stratigraphic plot of select pollen data (black dots) used to infer landscape openness through time. Grey shading shows the range boundaries (95% confidence interval) for datasets that we resampled at 2000-year intervals between 2 ka and 34 ka for correlation and volatility analysis, using the minimum and maximum ages calculated from age-depth modelling of the sequences (see Supplementary Text 3). Data plotted against climate anomalies (Hadley CM3) calculated for the region (20º N–11º S; 98º E–141º E) across the same period.

Supplementary Text 3: Methods for extracting and synthesizing time-transgressive pollen and isotopic records.

Mainland Sunda

NPK.2 – Nong Pa Kho (Thailand) (Penny, 2001)

Chronology: We remodeled five radiocarbon ages from analysis of bulk sediment samples reported in Penny (2001) using Bacon 2.5.0 (Blaauw and Christen, 2011) in R (R Core Team, 2023) (Fig. 4). We ran the model in 48 sections using a sediment accumulation rate (acc.mean) of 200 years cm^{-1} estimated from linear interpolation between adjacent date samples. We set an assumed age of -44 cal years before present (1994 CE) for the core top (0 cm). We set the basal depth for the core to 230 cm (d.max = 230). We used the Southern Hemisphere calibration curve (SH20) (Hogg et al., 2020), offset by −21 ± 6 yrs, in this analysis (see Hamilton et al. 2019a for justification of this curve and offset). We used the median age-depth model age output to estimate ages for the core sediments at 1-cm intervals.

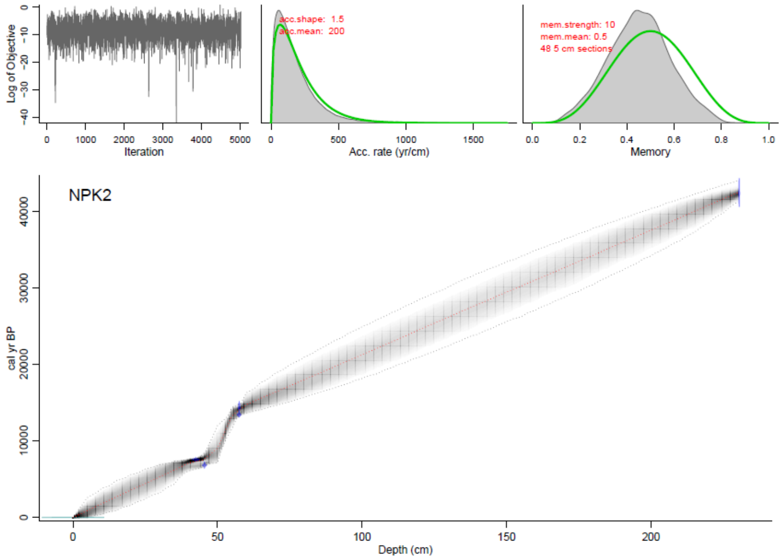

Fig. S4: Age-depth model produced for NPK2 (Penny, 2001).

Pollen data preparation: The author DP provided raw pollen data from the original study (Penny, 2001). We converted select data (grouped montane taxa and Poaceae) into dryland percentage (including Poaceae). Because this site comprises a large herbaceous wetland, much of the Poaceae data are likely derived from a local wetland source (Penny, 2001). This overemphasizes the 'openness' of the terrestrial vegetation signal.

Sunda – Insular Southeast Asia

SO18300 – Sunda shelf (South China Sea) (Wang et al., 2009)

Chronology: We remodeled five radiocarbon ages from analysis of organic samples (Hanebuth and Stattegger, 2004; Hanebuth et al., 2003) using `Bacon` 2.5.0 (Blaauw and Christen, 2011) in R (R Core Team, 2023) (Fig.. 5). We ran We ran the model in 179 sections using a sediment accumulation rate (acc.mean) of 50 years cm^{-1} estimated from linear interpolation between adjacent date samples. We set the basal depth for the core at 885 cm (d.max = 885) and the top of the core at d.min = 0. To maximise the probability of the dates overlapping with the age-depth model (60%), we did not assign a modern age to the upper-core sediments. We

used the marine calibration curve (Marine20) (Heaton et al., 2020) in this analysis. There are large uncertainties in the age-depth model produced for this core and the model rejected two out of the five dates (Fig. S5).

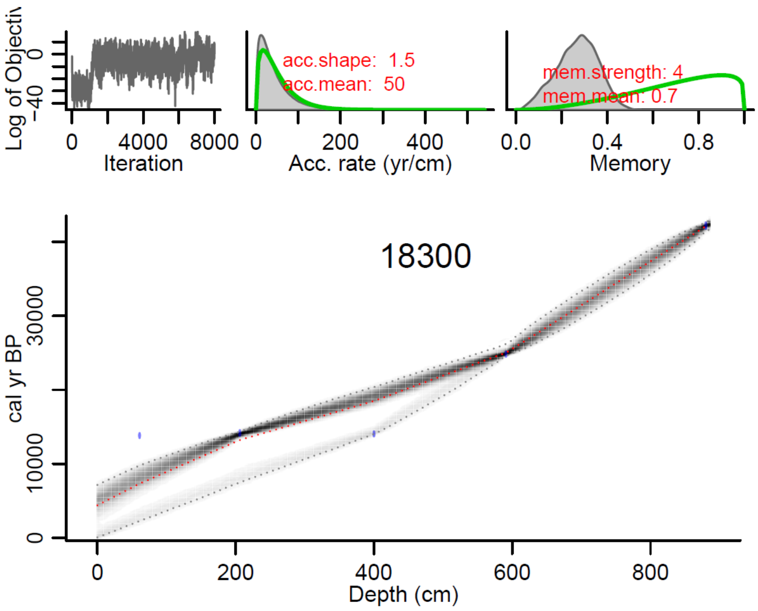

Fig. S5: Age-depth model produced for SO18300 (Wang et al., 2009).

Pollen data preparation: We extracted grass pollen data (% terrestrial land seed plants) from graphics presented in Wang et al. (2009) using WebPlotDigitizer (Rohatgi, 2021).

SO18323 – Sunda shelf (South China Sea) (Wang et al., 2009)

Chronology: We remodeled three radiocarbon ages, reported from analysis of mixed samples (Hanebuth and Stattegger, 2004; Hanebuth et al., 2003) for this study using Bacon 2.5.0 (Blaauw and Christen, 2011) in R (R Core Team, 2023) (Fig. S6). We ran the model in 110 sections using a sediment accumulation rate (acc.mean) of 50 years per cm estimated from linear interpolation between adjacent date samples. We set an assumed age of -47 cal yrs BP (1997 CE) was set for the core top (0 cm). The basal depth for the core was set at 540 cm (d.max = 540). We used the marine calibration curve (Marine20) (Heaton et al., 2020) in this analysis.

There are large uncertainties in the age-depth model produced for this core and the model rejected one out of the three dates used in the analyses (Fig. S6).

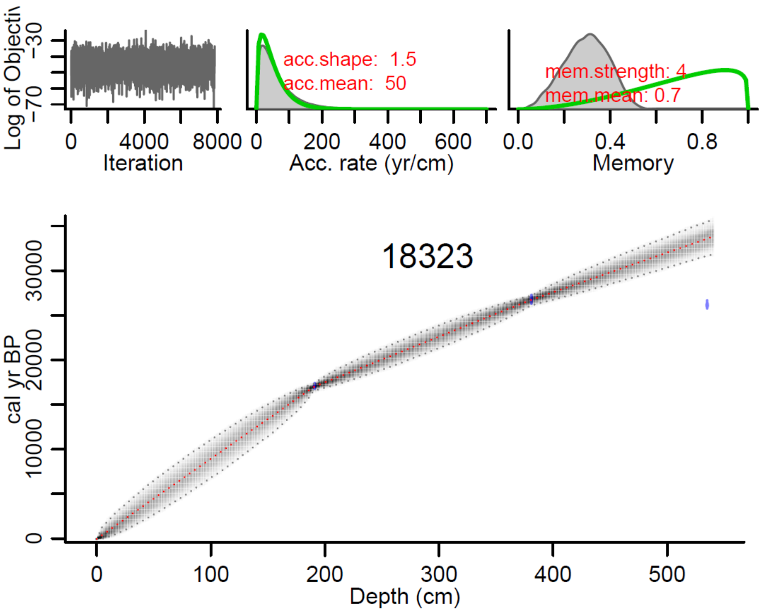

Fig. S6: Age-depth model produced for SO18323 (Wang et al., 2009).

Pollen data preparation: We extracted grass pollen data (% terrestrial land seed plants) from graphics presented in Wang et al. (2009) using WebPlotDigitizer (Rohatgi, 2021).

SO18302 – Sunda shelf (flat) (South China Sea) (Wang et al., 2009)

Chronology: We remodeled three radiocarbon ages, reported from analysis of mixed samples (Hanebuth and Stattegger, 2004; Hanebuth et al., 2003) using Bacon 2.5.0 (Blaauw and Christen, 2011) in R (R Core Team, 2023) (Fig. S 7). We ran the model in 121 sections using a sediment accumulation rate (acc.mean) of 20 years per cm estimated from linear interpolation between adjacent date samples. We set the basal depth for the core at 598 cm (d.max = 598) and the top of the core at d.min=0. To maximise the probability of the dates overlapping with the age-depth model (66%), the upper core sediments were not assumed to be modern. We used the marine calibration curve (Marine20) (Heaton et al., 2020) in this analysis.

There are large uncertainties in the age-depth model produced for this core and the model rejected one out of the three dates used in the analysis (Fig. S7).

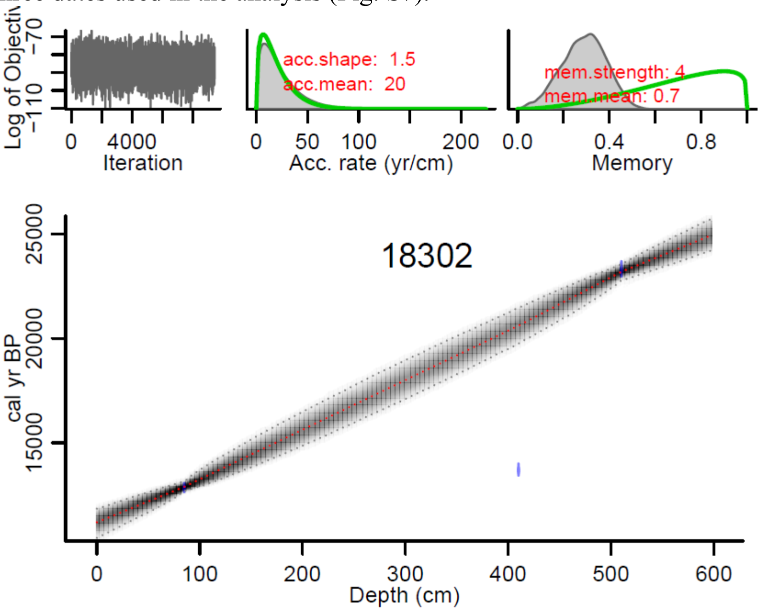

Fig. S7: Age-depth model produced for SO18302 (Wang et al., 2009).

Pollen data preparation: We extracted grass pollen data (% terrestrial land seed plants) from graphics presented in Wang et al. (2009) using WebPlotDigitizer (Rohatgi, 2021).

<u>17964 – Sunda shelf (slope) (South China Sea) (Sun et al., 2000)</u>

Chronology: We remodeled eight radiocarbon ages, reported from analysis of samples reported in Sun et al. (2000) for this study using Bacon 2.5.0 (Blaauw and Christen, 2011) in R (R Core Team, 2023) (Fig. S8). We ran the model in 131 sections using a sediment accumulation rate (acc.mean) of 20 years per cm estimated from linear interpolation between adjacent date samples. We used an assumed age of -45 cal yrs BP (1995 CE) for the core top (0 cm) and set 1303 cm as the basal depth for the core (d.max = 1303). We used the marine calibration curve (Marine20) (Heaton et al., 2020) fpr this analysis.

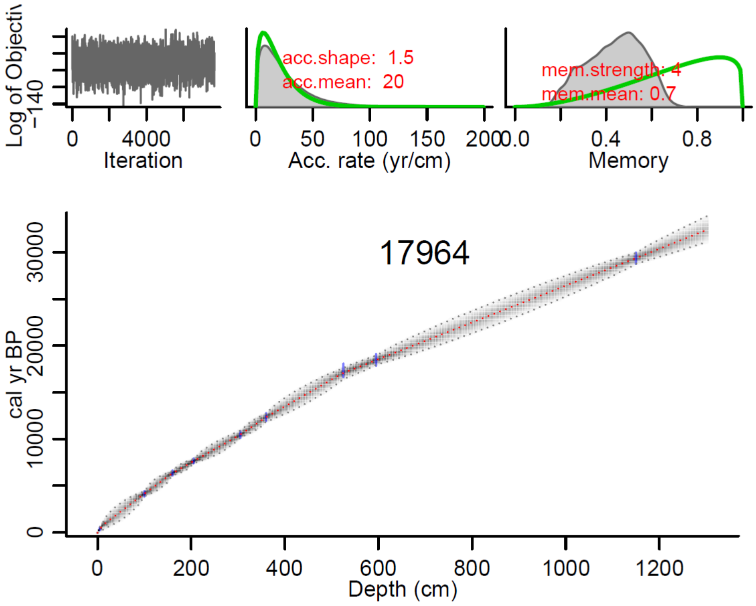

Fig. S8: Age-depth model produced for 17964 (Sun et al., 2000)

Pollen data preparation: Herb pollen (combined wetland and dryland herbs) and grouped arboreal pollen (% land seed plants) were extracted from graphics presented in (Sun et al., 2000) using WebPlotDigitizer.

<u>CB19 – Sunda shelf (South China Sea) (Yang et al., 2021)</u>

Chronology: We remodeled eight radiocarbon ages, reported from analysis of seven mixed planktonic foraminifera/ *Neogloboquadrina dutertrei* samples in Yang et al. (2021) using Bacon 2.5.0 (Blaauw and Christen, 2011) in R (R Core Team, 2023) (Fig. S9). We ran the model in 70 sections using a sediment accumulation rate (acc.mean) of 100 years per cm estimated from linear interpolation between adjacent date samples. We set an assumed age of -62 cal yrs BP (2012 CE) for the core top (0 cm) and set the basal depth for the core at 342 cm (d.max = 342). We used the marine calibration curve (Marine20) (Heaton et al., 2020) in this analysis.

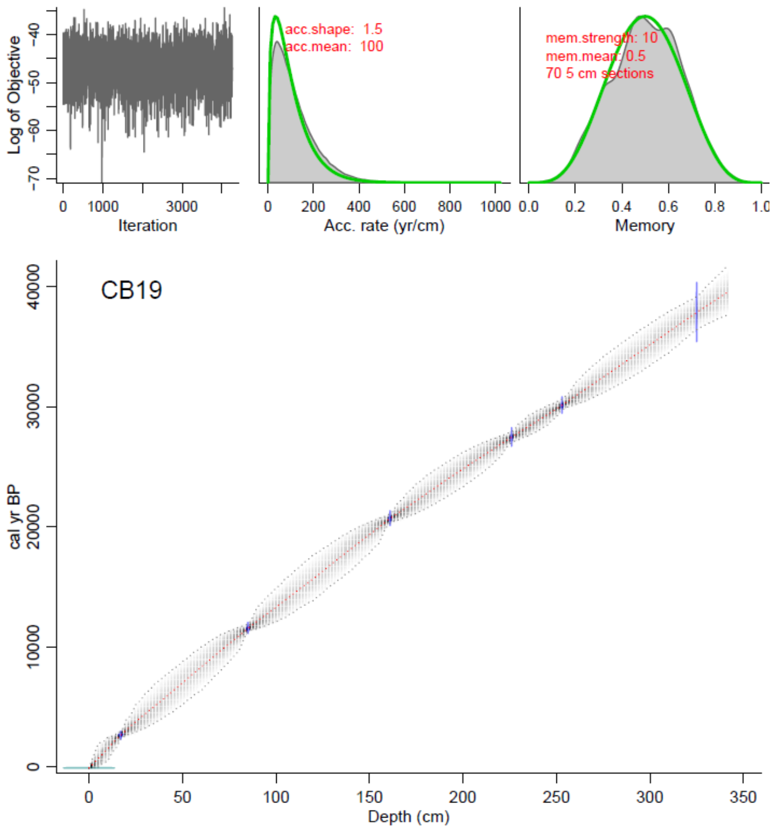

Fig. S9: Age-depth model produced for CB-19 (Yang et al., 2021).

Pollen data preparation: We extracted grouped montane forest pollen (i.e., the sum of upper and lower montane types) and grass pollen (% terrestrial plants) from the graphics presented in Yang et al. (2021) using WebPlotDigitizer (Rohatgi, 2021).

NS07-25 – South China Sea (Nansha Trough) (Xiang et al., 2009)

Chronology: We remodeled eleven radiocarbon ages reported from planktonic foraminifera in Xiang et al. (2009) using Bacon 2.5.0 (Blaauw and Christen, 2011) in R (R Core Team, 2023) (Fig. S10). We ran the model in 113 sections using the marine calibration curve (Marine20) (Heaton et al., 2020). We used a sediment accumulation rate (acc.mean) of 50 years per cm estimated from linear interpolation between adjacent date samples. We set the basal depth for the core at 556 cm (d.max = 556), the minimum core depth at 0cm (d.min = 0) and used an assumed age of -57 cal. BP (2007 CE) for the core top (0 cm).

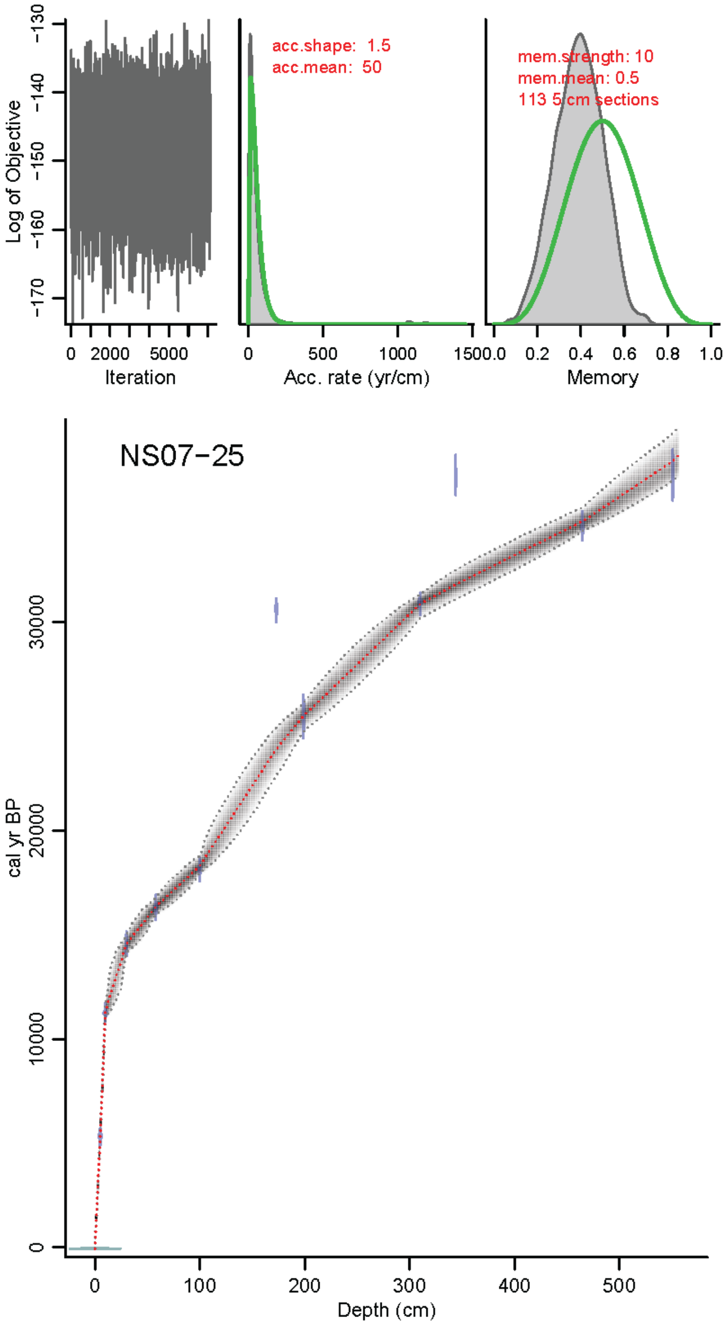

Fig. S10: Age-depth model produced for NS07-25 (Xiang et al., 2009)

Pollen data preparation: Thilakanayaka et al. (2019) provided the raw pollen data from the original study on request. We calculated grass and grouped montane forest pollen as a percent of the dryland pollen sum for this study.

PemCoreB – Lake Pemerak Core B (West Kalimantan, Indonesia [Borneo]) (Anshari et al., 2004)

Chronology: We remodeled eight radiocarbon ages for Lake Pemerak Core B (West Kalimantan, Borneo) reported from 7 x pollen concentrates and 1 x bulk sediment sample (Anshari et al., 2004) using Bacon 2.5.0 (Blaauw and Christen, 2011) in R (R Core Team, 2023) (Fig. S11). An hiatus was inserted at the sediment boundary at 69cm, where extrapolation between dated points indicate a ~10,000 year time gap over a depth interval of 4 cm. We set the sedimentation rate between 0 and 69 cm at 50 years per cm, and the sedimentation rate between 69 cm and the basal depth (120 cm) at 200 years per cm based on linear extrapolation between dated points on either side of the sediment boundary. We ran the model in 25 sections using the Southern Hemisphere calibration curve (SHCal20) (Hogg et al., 2020). We set basal depth for the core at 120 cm (d.max = 120), and set the minimum core depth at 0cm (d.min = 0).

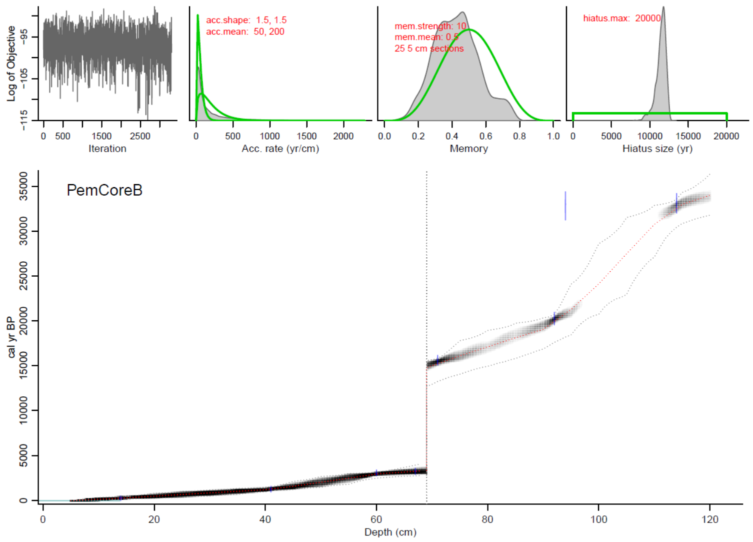

Fig. S11: Age-depth model produced for PemB (Anshari et al., 2004).

Pollen data preparation: We extracted percentage pollen data grouped into major plant functional types from the original publication using WebPlotDigitizer (Rohatgi, 2021). The plots for herbs (%) and montane forest (%) were used in the analysis.

<u>PemCoreC – Lake Pemerak Core C (West Kalimantan, Indonesia [Borneo]) (Anshari et al., 2004)</u>

Chronology: We remodeled three radiocarbon ages for Lake Pemerak Core C (West Kalimantan, Borneo) reported from pollen concentrates (Anshari et al., 2004) using Bacon 2.5.0 (Blaauw and Christen, 2011) in R (R Core Team, 2023) (Fig. S12). We ran the model in 62 sections using the Southern Hemisphere calibration curve (SHCal20) (Hogg et al., 2020) and a sediment accumulation rate (acc.mean) of 500 years per cm estimated from linear interpolation between adjacent date samples. We set the basal depth for the core at 300 cm (d.max = 300), and the minimum core depth at 0cm (d.min = 0), noting that the core surface sediments are unlikely to be modern. Because the oldest dated point was 81 cm depth, data from this core is only presented to 80 cm depth (modelled at 44901 cal. BP).

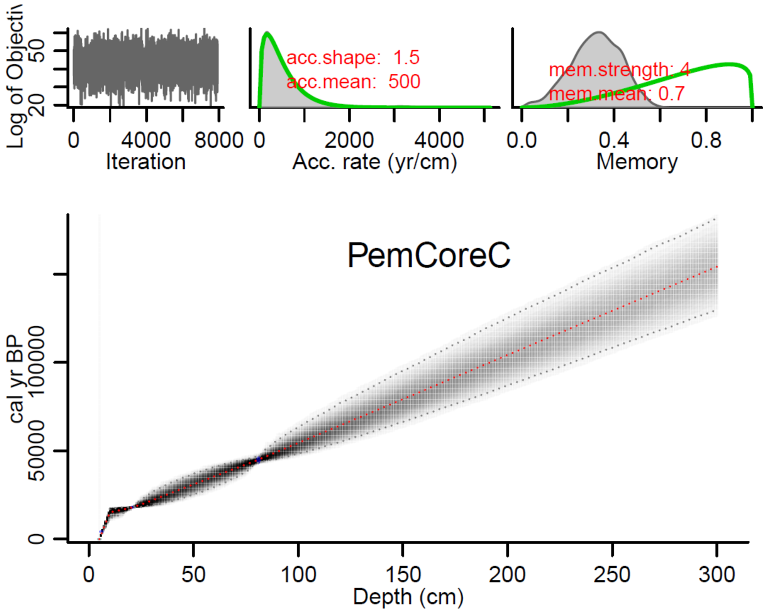

Fig. S12: Age-depth model produced for PemC (Anshari et al., 2004).

Pollen data preparation: We extracted percentage pollen data grouped into major plant functional types from graphics presented in Anshari et al. (2004) using WebPlotDigitizer (Rohatgi, 2021). We used the plots for herbs (%) and montane forest (%) in this analysis.

PemCoreD – Lake Pemerak Core D (West Kalimantan, Indonesia [Borneo]) (Anshari et al., 2004)

Chronology: We remodeled six radiocarbon ages for Lake Pemerak Core D (West Kalimantan, Borneo) reported from bulk sediment and pollen concentrates (Anshari et al., 2004) using Bacon 2.5.0 (Blaauw and Christen, 2011) in R (R Core Team, 2023) (Fig. S13). We ran the model in 22 sections using the Southern Hemisphere calibration curve (SHCal20) (Hogg et al., 2020) and a sediment accumulation rate (acc.mean) of 200 years per cm estimated from linear interpolation between adjacent date samples. Due to the poor quality of the chronological data, no age was set for the core top sediments (0 cm). We set the basal depth for the core at 100 cm (d.max = 100).

The chronological model for this core is very poor, with two ages rejected from the final age depth model (WK5775 at 53cm depth and OZE143 at 61cm depth), and a very wide range of error calculated for the younger portion of the core sediments (Fig. S13).

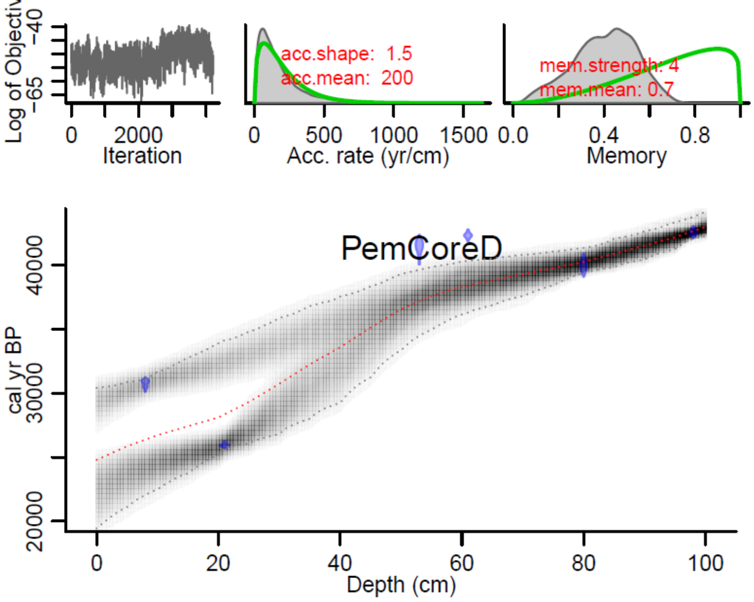

Fig. S13: Age-depth model produced for PemD (Anshari et al., 2004).

Pollen data preparation: We extracted percentage pollen data grouped into major plant functional types from graphics presented in Anshari et al. (2004) using WebPlotDigitizer (Rohatgi, 2021). We used the plots for herbs (%) and montane forest (%) in this analysis.

Saleh Cave – Kalimantan, Indonesia (Borneo) (Wurster et al., 2019)

Chronology: We used the original chronology (Wurster et al., 2019) in this analysis as extractable data are only plotted against age rather than depth.

Isotopic data preparation: We extracted $\delta^{13}C_{guano}$ data from graphics in Wurster et al. (2019) using WebPlotDigitizer (Rohatgi, 2021).

Batu Cave – Sarawak, Malaysia (Borneo) (Wurster et al., 2010)

Chronology: We used the original chronology (Wurster et al., 2010) in this analysis as extractable data are only plotted against age rather than depth.

Isotopic data preparation: We extracted $\delta^{13}C_{guano}$ data from graphics in Wurster et al. (2010) using WebPlotDigitizer (Rohatgi, 2021).

BAR94-42 (Indian Ocean off Southwest Sumatra) (van der Kaars et al., 2010)

Chronology: We used the original chronology (van der Kaars et al., 2010) in this analysis as extractable data are only plotted against age rather than depth.

Pollen data preparation: We extracted dryland percentage data (grouped lowland forest, montane forest, and herb pollen) from van der Kaars et al. (2010) using WebPlotDigitizer (Rohatgi, 2021) for use in this study.

SO189-144KL – Nias Basin (Indian Ocean off Southwest Sumatra) (Niedermeyer et al., 2014a)

Chronology: We remodeled thirty-six radiocarbon ages for SO189-144KL produced from analysis of mixed planktonic, *G. sacculifer* and *G. ruber* samples (Mohtadi et al., 2014) for this study using Bacon 2.5.0 (Blaauw and Christen, 2011) in R (R Core Team, 2023) (Fig. S14). We ran the model in 166 sections using the marine calibration curve (Marine20) (Heaton et al., 2020) and a sediment accumulation rate (acc.mean) of 20 years per cm estimated from linear interpolation between adjacent date samples. We set an assumed age of -56 cal yrs BP (2006 CE) for the core top (0 cm) and set the basal depth at 823 cm (d.max = 823).

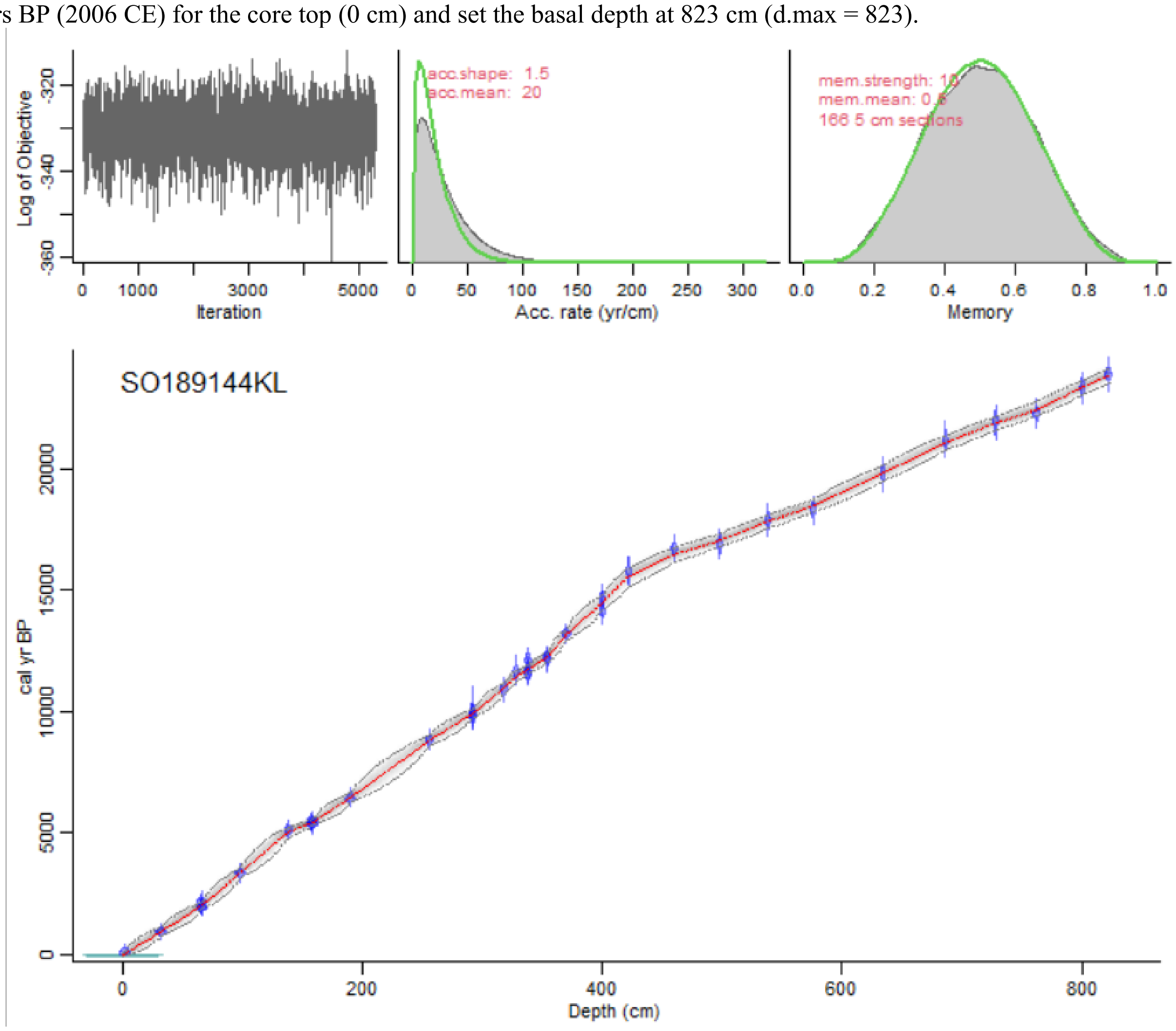

Fig. S14: Age-depth model produced for SO189-144KL (Mohtadi et al., 2011b).

Isotopic data preparation: We obtained $\delta^{13}C$ C30AA data from the PANGEA database (Niedermeyer et al., 2014b).

Pea-Sim-sim (PSS) (North Sumatra, Indonesia) (Maloney, 1980)

Chronology: We remodeled thirteen radiocarbon ages for Pea-Sim-sim Swamp for this study using Bacon 2.5.0 (Blaauw and Christen, 2011) in R (R Core Team, 2023) (Fig. S15). We estimated depths from the pollen diagram in Maloney (1980) (Table S2). We ran the model in 197 sections using the Southern Hemisphere calibration curve (SHCal20) (Hogg et al., 2020) and a sediment accumulation rate (acc.mean) of 20 years per cm estimated from linear interpolation between adjacent date samples. We set an assumed age of -30 cal yrs BP (1980 CE) for the core top (0 cm) and set the basal depth at 975 cm (d.max = 975).

Table S2: 14C ages and depths estimated from pollen diagram presented in Maloney (1980)

^{14}C age	error	depth (cm)
5000	130	145
7280	180	200
8049	80	215
8230	150	235
9840	125	325
11494	75	415
12500	140	500
13500	180	595
15818	100	665
16212	160	715
17722	75	820
17880	200	885
18497	95	975

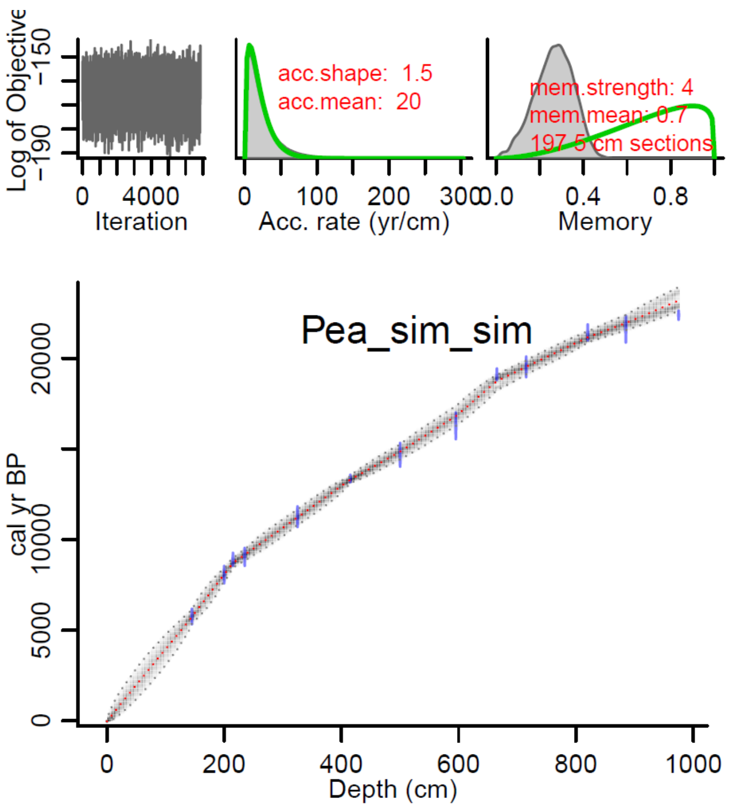

Fig. S15: Age-depth model produced for Pea sim sim (Maloney, 1980).

Pollen data preparation: We extracted grouped forest percentage data (% of the dryland sum) from graphics presented in Maloney (1980) using WebPlotDigitizer (Rohatgi, 2021).

Mbelen Cave (North Sumatra) (McCarthy et al., 2022)

Chronology: We remodeled eight radiocarbon ages from solvent-extracted, base-acid guano (SEBAG) in McCarthy et al. (2022) for this study using Bacon 2.5.0 (Blaauw and Christen, 2011) in R (R Core Team, 2023) (Fig. S16). We ran the model in 51 sections using the Northern Hemisphere calibration curve (IntCal20) (Hogg et al., 2020) and used a sediment accumulation rate (acc.mean) of 50 years per cm estimated from linear

interpolation between adjacent date samples. We set the surface and basal depth for the cave sediments at 0 and 280 cm, respectively (d.min=0, d.max = 280).

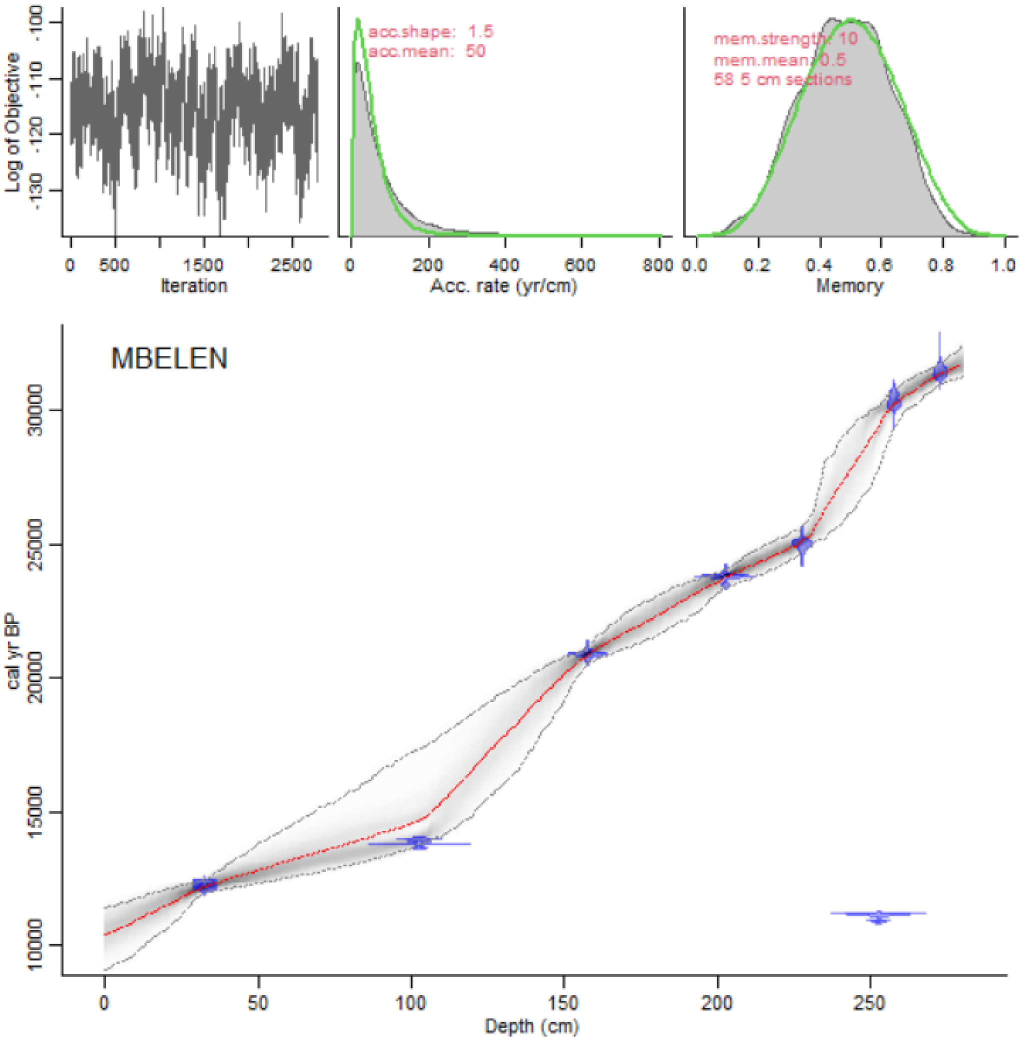

Fig. S16: Age-depth model produced for Mbelen Cave (McCarthy et al., 2022)

Isotopic data preparation: $\delta^{13}C_{guano}$ data provided with the original paper were used in this analysis.

<u>DDA- Danau di Atas swamp (Sumatra) (Newsome and Flenley, 1988)</u>

Chronology: We remodeled ten radiocarbon ages from DDA bulk sediment samples (Newsome and Flenley, 1988) for this study using Bacon 2.5.0 (Blaauw and Christen, 2011) in R (R Core Team, 2023) (Fig. S17). We ran the model in 169 sections using the Northern Hemisphere calibration curve (IntCal20) (Hogg et al., 2020) and a sediment accumulation rate (acc.mean) of 20 years per cm estimated from linear interpolation between adjacent date samples. We set an assumed age of -27 cal yrs BP (1977 CE) for the core top (0 cm) and set the basal depth at 1650 cm (d.max = 1650).

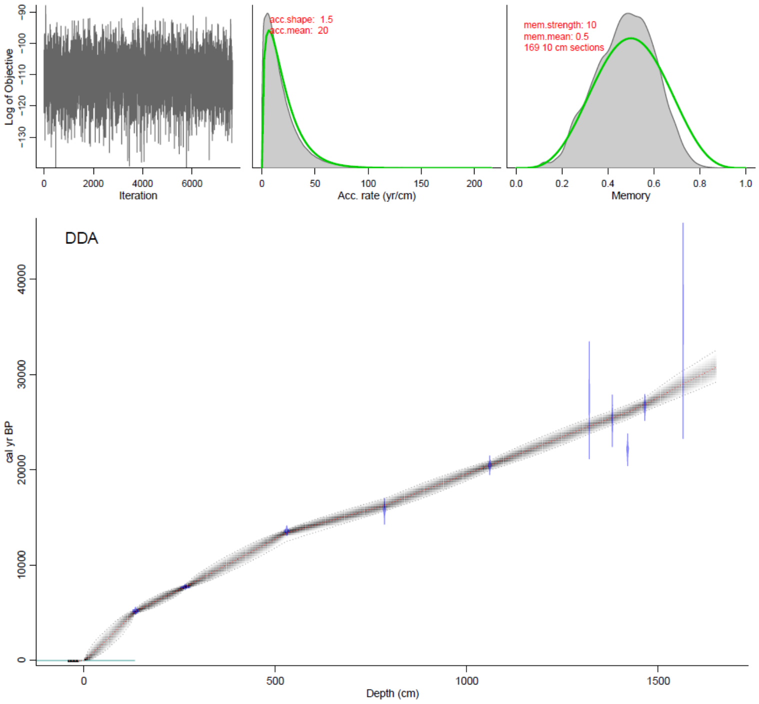

Fig. S17: Age-depth model produced for DDA (Newsome and Flenley, 1988).

Pollen data preparation: We extracted Poaceae pollen data (% of the dryland sum) from Newsome and Flenley (1988) using WebPlotDigitizer (Rohatgi, 2021).

PeaBullok A (PB-A) - Pea Bullok (Sumatra) (Maloney and McCormac, 1995)

Chronology: We remodeled seven radiocarbon ages from PeaBullokA bulk sediment samples (Maloney and McCormac, 1995) using Bacon 2.5.0 (Blaauw and Christen, 2011) in R (R Core Team, 2023) (Fig. S18). We ran the model in 162 sections using the Southern Hemisphere calibration curve (SHCal20) (Hogg et al., 2020) and a sediment accumulation rate (acc.mean) of 50 years per cm estimated from linear interpolation between adjacent date samples. We set an assumed age of -40 cal yrs BP (1990 CE) for the core top (0 cm) and set the basal depth for the core at 800 cm (d.max = 800).

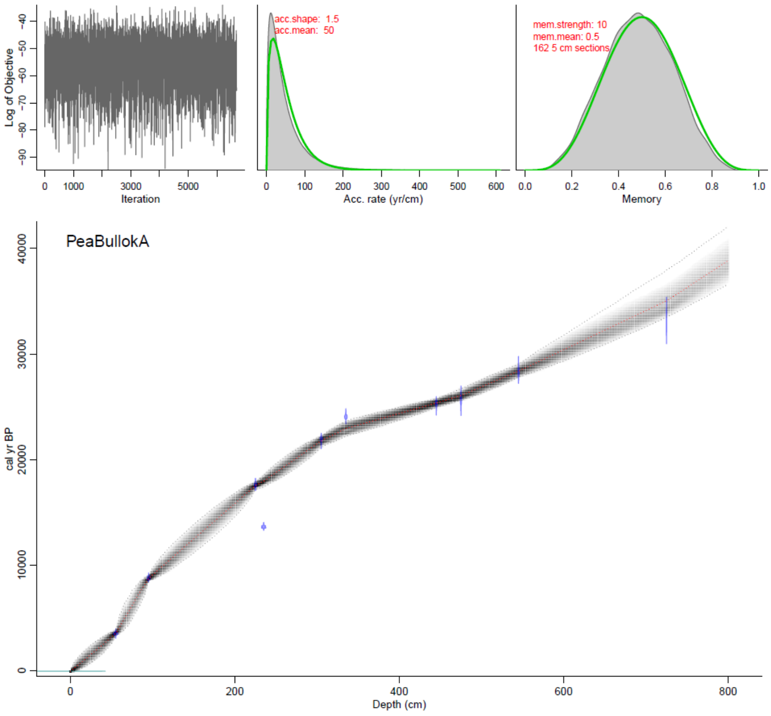

Fig. S18: Age-depth model produced for PeaBullokA (Maloney and McCormac, 1995).

Pollen data preparation: We extracted Poaceae pollen data (% of the dryland sum) from Maloney and McCormac, (1995) using WebPlotDigitizer (Rohatgi, 2021).

GeoB10053-7 (Java Sea) (Ruan et al., 2018a, b, c, 2019)

Chronology: We remodeled nineteen radiocarbon ages from Mohtadi et al. (2011a) using Bacon 2.5.0 (Blaauw and Christen, 2011) in R (R Core Team, 2023) (Fig. S19). We ran the model in 158 sections using a sediment accumulation rate (acc.mean) of 20 years per cm estimated from linear interpolation between adjacent date samples. We set the core top sediments to -55 cal. BP and used the marine calibration curve (Marine20) (Heaton et al., 2020).

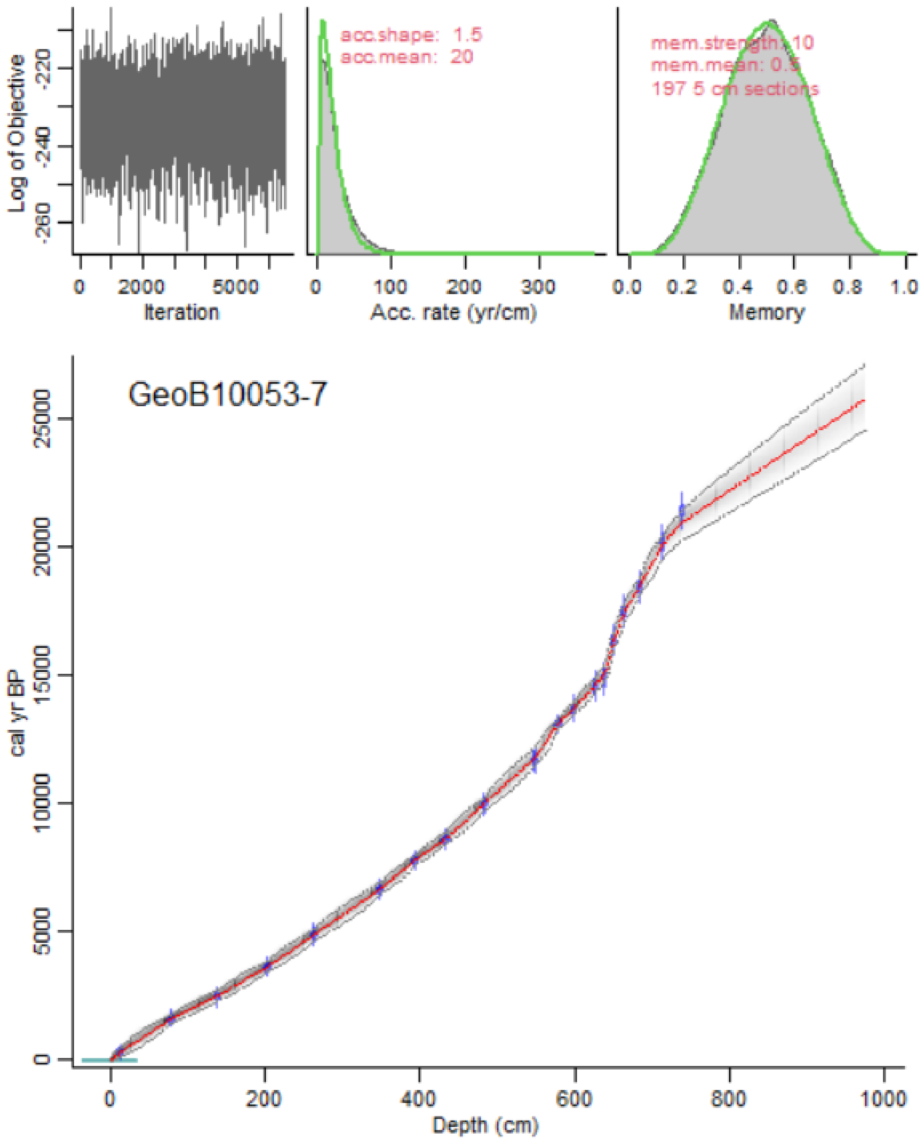

Fig. S19: Age-depth model produced for GeoB10053-7 (Ruan et al., 2018c).

Isotopic data: We obtained $\delta^{13}C$ data from the PANGEA database (Ruan et al., 2018b).

Pollen data preparation: We obtained grass, montane forest and lowland forest pollen percentage data from the PANGEA database (Ruan et al., 2018b).

Sunda – Palawan

MC1– Philippines - Palawan (Makangit Cave) (Bird et al., 2007)

Chronology: We remodeled two radiocarbon dates from guano (Bird et al., 2007) using Bacon 2.5.0 (Blaauw and Christen, 2011) in R (R Core Team, 2023) (Fig. S20). We ran the model in 172 sections using the Northern Hemisphere calibration curve (IntCal20) and a sediment accumulation rate (acc.mean) of 500 years per cm estimated from linear interpolation between adjacent date samples. Given the very low number of sampling points, no assumed core-top age was set for this sequence. We set the basal depth for the core at 180 cm (d.max = 180).

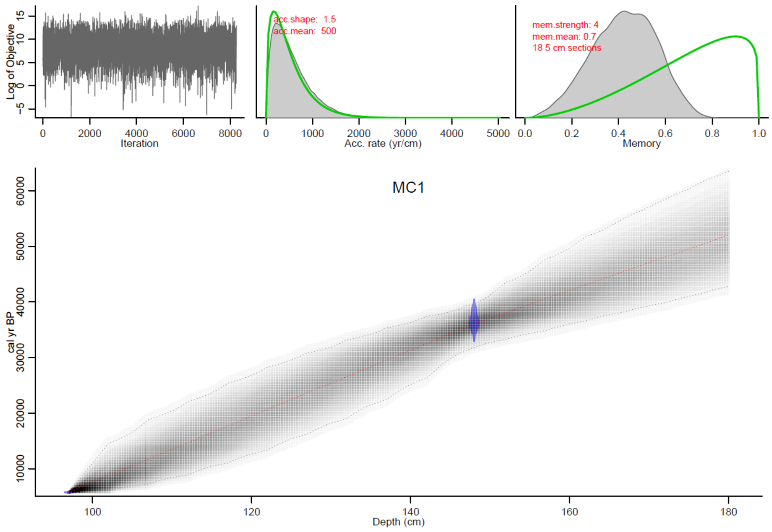

Fig. S20: Age-depth model produced for MC1 (Bird et al., 2007).

Isotopic data preparation: We extracted $\delta^{13}C_{guano}$ from Bird et al. (2007) using WebPlotDigitizer (Rohatgi, 2021).

Batu Cave – Philippines (Palawan) (Wurster et al., 2010)

Chronology: We used the original chronology (Wurster et al., 2010) in this analysis as extractable data are only plotted against age rather than depth.

Isotopic data preparation: We extracted $\delta^{13}C_{guano}$ data from Wurster et al. (2010) using WebPlotDigitizer (Rohatgi, 2021).

Gangub Cave – Philippines (Palawan) (Wurster et al., 2010)

Chronology: We used the original chronology (Wurster et al., 2010) in this analysis as extractable data are only plotted against age rather than depth.

Isotopic data preparation: We extracted $\delta^{13}C_{guano}$ data from Wurster et al. (2010) using WebPlotDigitizer (Rohatgi, 2021).

The Philippines (excluding Palawan)

MD06-3075 – Philippines (Davao Bay) (Bian et al., 2011) (extrapolated from MD982181 (Stott et al., 2002; Stott, 2007))

Chronology: The original chronology for MD06-3075 was produced by pairing planktonic foraminifera δ18O records produced for MD06-3075 with that of MD98-2181 (6.3°N, 125.83°E) – the latter of which is well dated using 42 AMS ^{14}C dates (Stott et al., 2002; Stott, 2007). We remodeled the dates from MD98-2181 using Bacon 2.5.0 (Blaauw and Christen, 2011) in R (R Core Team, 2023) (Fig. S21). We ran the model in 18 sections using the marine calibration curve (Marine20) (Heaton et al., 2020) and a sediment accumulation rate (acc.mean) of 500 years per cm estimated from linear interpolation between adjacent date samples. We set an assumed age of -50 cal yrs BP for the core top (0 cm) and set the basal depth for the core at 1711 cm (d.max = 1711). Five tied depth points between MD06-3075 and MD98-2181 were paired to produce an age-depth model for MD06-3075.

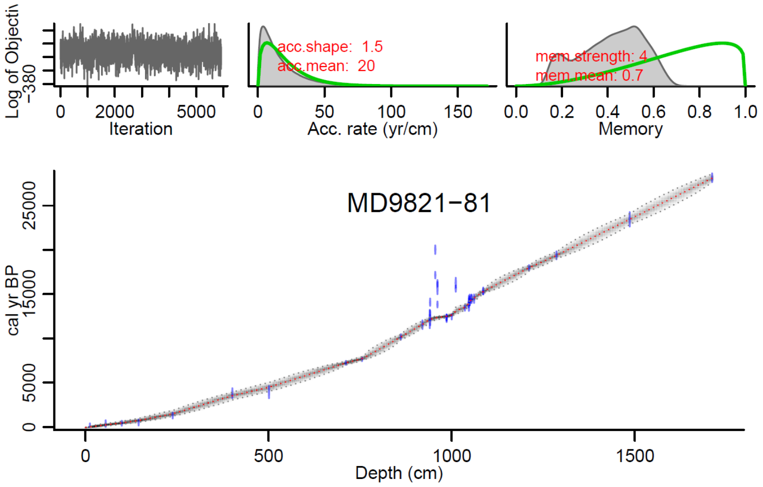

Fig. S21: Age-depth model produced for MD982181 (Stott et al., 2002; Stott, 2007) that was paired with MD06-3075 (Bian et al., 2011).

Pollen data preparation: We extracted grouped montane forest, mangrove forest, lowland forest, and grassland pollen percentage data from Bian et al. (2011) using WebPlotDigitizer (Rohatgi, 2021). These data were recalculated to reflect percentage of the dryland sum by removing the mangrove data.

Wallacea

GeoB10069-3 – Suvu Sea (Sumba) (Dubois et al., 2014)

Chronology: We remodeled eighteen radiocarbon ages and their offset values reported from mixed planktonic samples (Dubois, 2014) using Bacon 2.5.0 (Blaauw and Christen, 2011) in R (R Core Team, 2023) (Fig. S22). We ran the model in 192 sections with a boundary set at 725cm. We used a sediment accumulation rate (acc.mean) of 20 years per cm for sediments shallower than 725cm, and 100 years per cm for sediments deeper than 725cm based off linear interpolation between adjacent date samples. We set the core top sediments were set to -50 cal. BP and used the marine calibration curve (Marine20) (Heaton et al., 2020).

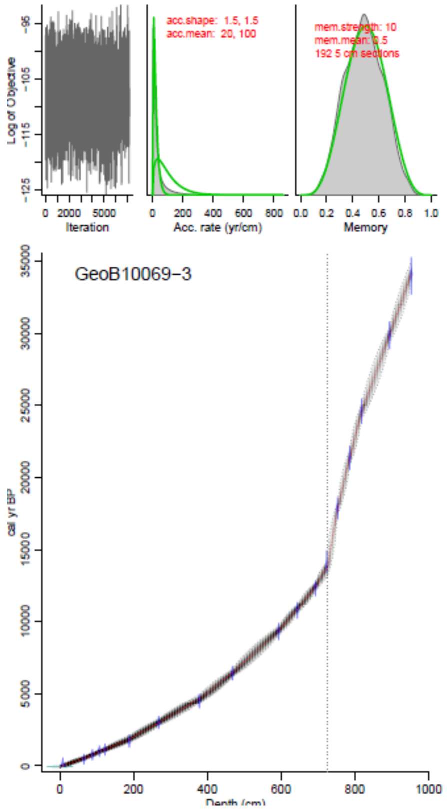

Fig. S22: Age-depth model produced for GeoB10069-3 (Dubois et al., 2014).

Isotopic data: We sourced δ13C-C30FA data (‰) used in Dubois et al. (2014) from the NOAA database.

Pollen data preparation: We extracted grouped percentage pollen data (monsoonal (seasonal) forest, montane forest, and C4 herbs) from Dubois et al. (2014) using WebPlotDigitizer (Rohatgi, 2021).

TOW10-9B (TOW9) – Towuti (Sulawesi) (Russell et al., 2014)

Chronology: We remodeled twenty-three reservoir-corrected radiocarbon ages reported from analysis of 20 bulk organic carbon and three terrestrial macrofossil samples and one Pb^{210} age (Russell et al., 2014) using Bacon 2.5.0 (Blaauw and Christen, 2011) in R (R Core Team, 2023) (Fig. S23). We ran the model in 123 sections using a sediment accumulation rate (acc.mean) of 50 years per cm and used the Southern Hemisphere calibration curve (SHCal20) (Hogg et al., 2020). We set the core top sediments to -64cal. BP, and the basal sediments were set to 1156cm (d.max=1156).

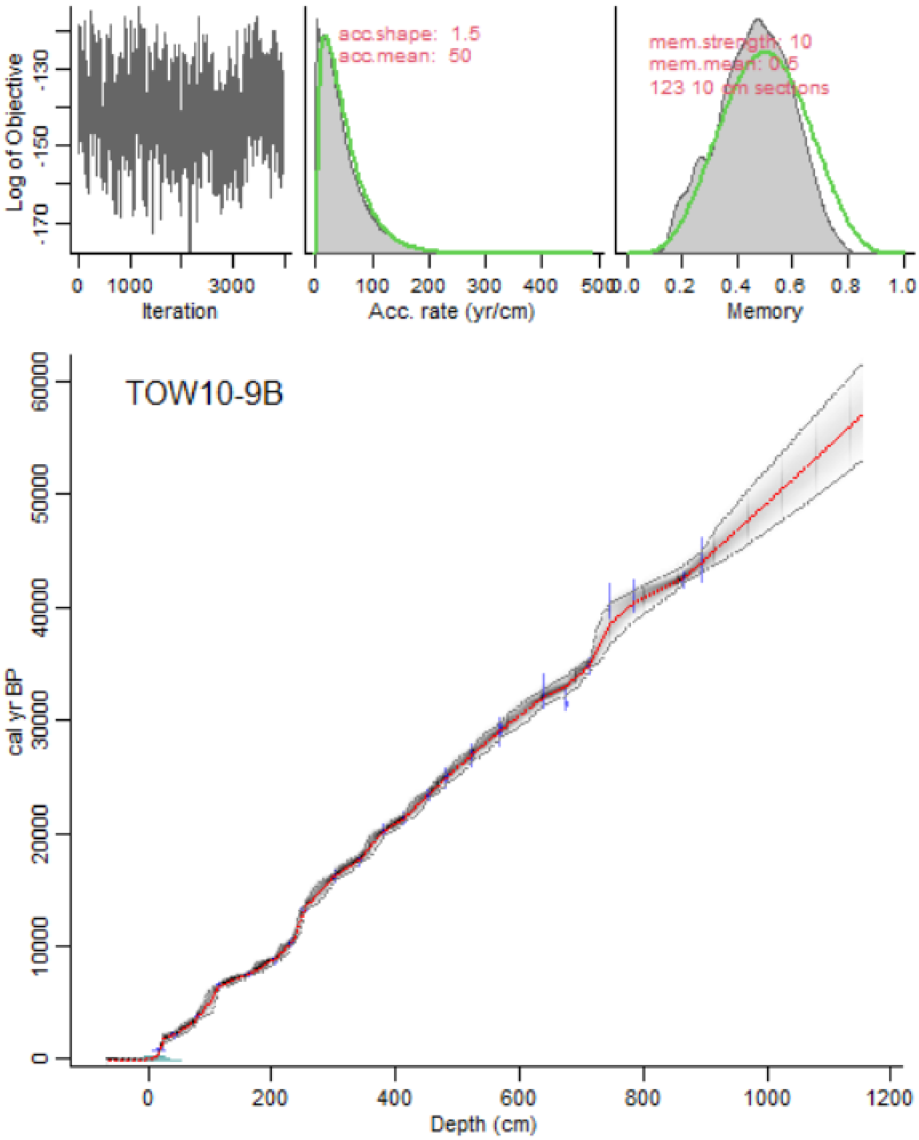

Fig. S23: Age-depth model produced for TOW9 (Russell et al., 2014)

Isotopic data preparation: We sourced $\delta^{13}C_{leafwaxC28}$ data (‰) used in Russell et al. (2014) from the NOAA database.

IDLE-MAT10-2B (MAT10-2B) – Matano (Sulawesi) (Wicaksono et al., 2015)

Chronology: We remodeled ten reservoir-corrected radiocarbon ages reported from analysis of bulk sediment and wood/plant samples (Wicaksono et al., 2015) using Bacon 2.5.0 (Blaauw and Christen, 2011) in R (R Core Team, 2023) (Fig. S24). We ran the model in 183 sections using the Southern Hemisphere calibration curve (SHCal20) (Hogg et al., 2020) and a sediment accumulation rate (acc.mean) of 50 years per cm estimated from linear interpolation between adjacent date samples. We set an assumed age of -60 cal yrs BP for the core top (0 cm) and set the basal depth at 907 cm (d.max = 907).

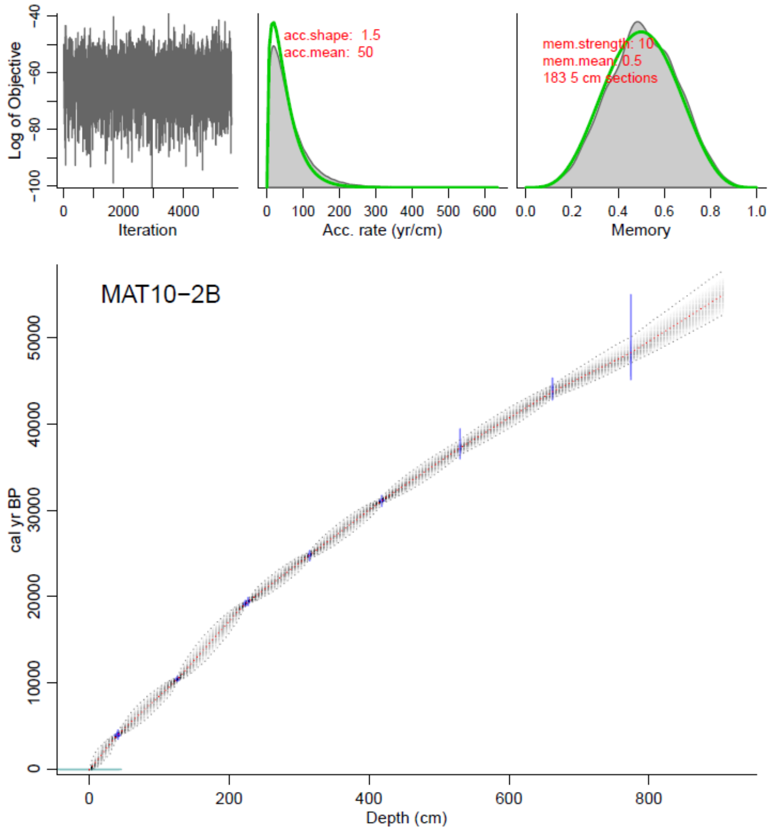

Fig. S24: Age-depth model produced for MAT10-2B (Wicaksono et al., 2015).

Isotopic data preparation: We extracted $\delta^{13}C_{leafwaxC28}$ from the original study using WebPlotDigitizer (Rohatgi, 2021).

SO18515 –Mandar Bay (Sulawesi) (Wicaksono et al., 2017)

Chronology: We used the original chronology in Wicaksono et al. (2017) as extractable data are only plotted against age rather than depth.

Isotopic data preparation: We extracted $\delta^{13}C_{leafwaxC28}$ from the original study using WebPlotDigitizer (Rohatgi, 2021).

T1-A/B – Lake Tondano (Sulawesi) (Dam et al., 2001)

Chronology: We remodeled eight radiocarbon ages reported from analysis of bulk sediment samples (Dam et al., 2001) using Bacon 2.5.0 (Blaauw and Christen, 2011) in R (R Core Team, 2023) (Fig. S25). We ran the model in 146 sections using the Northern Hemisphere calibration curve (IntCal20) (Hogg et al., 2020) and a sediment accumulation rate (acc.mean) of 10 years per cm estimated from linear interpolation between adjacent date samples. We set an age of 0±30 cal yrs BP (1950 CE) for the sediments at 95cm depth given that these returned a modern age estimate upon ^{14}C analysis. An assumed age of -45 cal yrs BP (1995 CE) was set for the core top (0 cm), and the basal depth was set at 1450 cm (d.max = 1450). We included an hiatus for the sediments at 1050 cm (hiatus.depth = 1050) per the original age-depth interpretation (Dam et al., 2001), which was modelled as spanning 33596 to 17423 cal yrs BP (median age). Two samples were outliers in the age depth model (105800±80 at 315 cm; 11830±440 at 1215 cm, the latter of which was excluded from the plot) (Fig. S25).

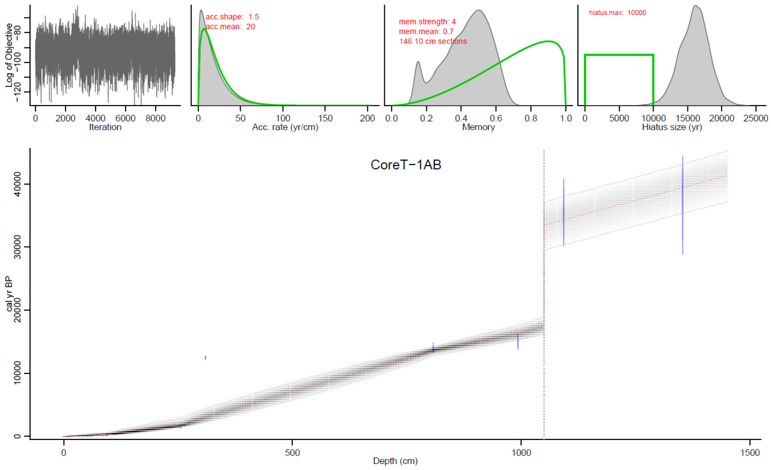

Fig. S25: Age-depth model produced for Core T-1AB (Dam et al., 2001).

Pollen data preparation: We extracted grass pollen percentage data as a percentage of the dryland count from Dam et al. (2001) using WebPlotDigitizer (Rohatgi, 2021).

G4K12P1 (K12P1) – Molucca Sea Core (Maluku) (van der Kaars, 1991)

Chronology: We remodeled two radiocarbon ages for the core, reported from analysis of Pteropods in van der Kaars (1991) using Bacon 2.5.0 (Blaauw and Christen, 2011) in R (R Core Team, 2023) (Fig. S26). We ran the model in 109 sections using the marine calibration curve (Marine20) (Heaton et al., 2020) and a sediment accumulation rate (acc.mean) of 50 years per cm estimated from linear interpolation between adjacent date samples. We set an assumed age of -40 cal yrs BP for the core top (0 cm) and set the basal depth at 535 cm (d.max = 535).

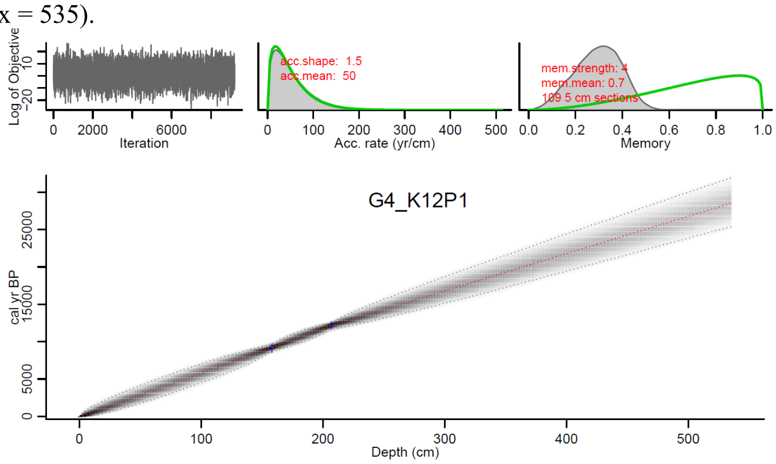

Fig. S26: Age-depth model produced for G4K12P1 (van der Kaars, 1991).

Pollen data preparation: We extracted grouped montane forest, mangrove forest lowland forest, and woodland and grass pollen percentage data from van der Kaars (1991) using WebPlotDigitizer (Rohatgi, 2021).

BJ8-03-91GGC (91GGC) – Celebes Sea – (Dubois et al., 2014)

Chronology: We remodeled six radiocarbon ages for the core from the analysis of mixed planktonics and *G. sacculifer* (Dubois, 2014) using Bacon 2.5.0 (Blaauw and Christen, 2011) in R (R Core Team, 2023) (Fig. S27). We ran the model in 77 sections using the marine calibration curve (Marine20) (Heaton et al., 2020) and a sediment accumulation rate (acc.mean) of 50 years per cm estimated from linear interpolation between adjacent date samples. We set an assumed age of -41 cal yrs BP for the core top (0 cm), and set the basal depth at 375 cm (d.max = 375).

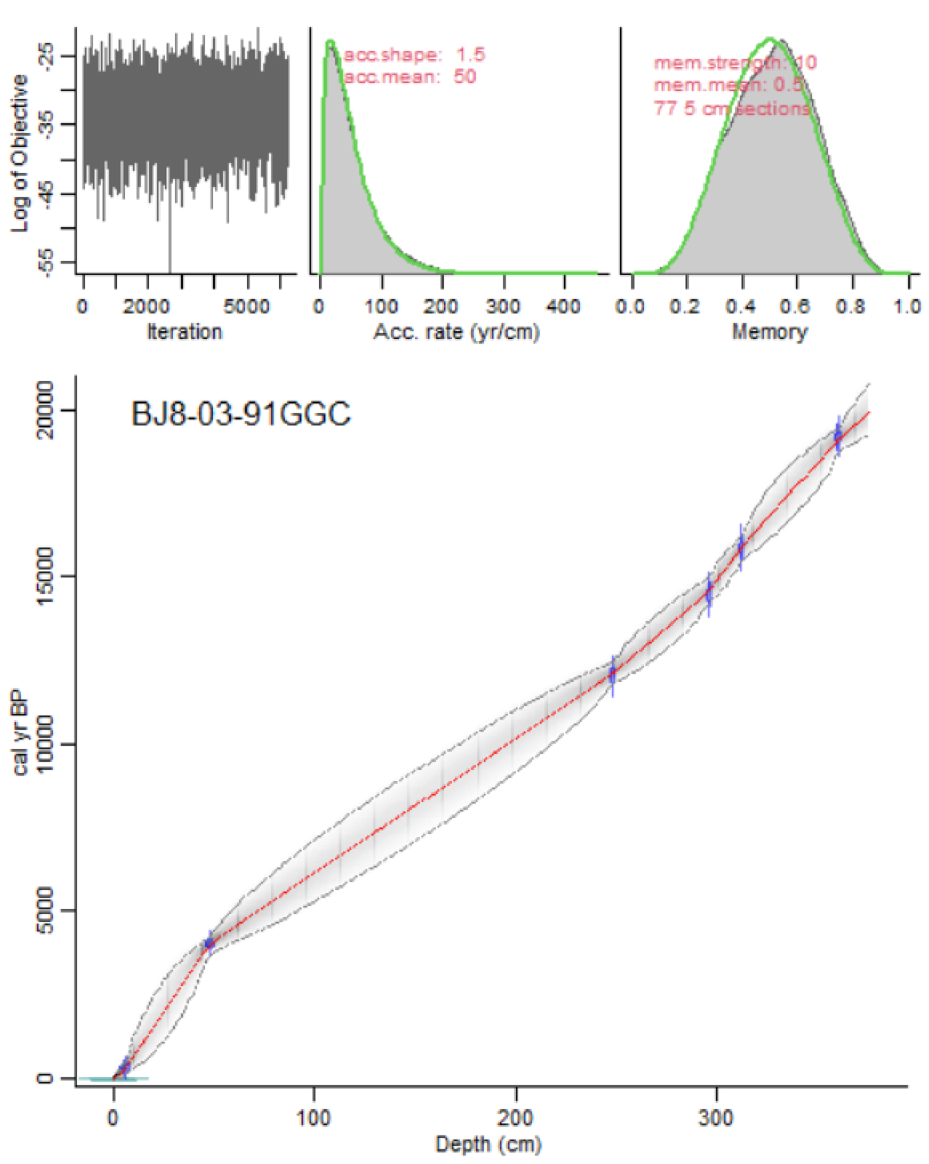

Fig. S27: Age-depth model produced for BJ8-03-91GGC (Dubois et al., 2014)

Isotopic data: We obtained $\delta^{13}C$ data (Dubois, 2014) from the NOAA database.

Sahul

Hordorli – Danau Hordorli – (West New Guinea) (Hope and Tulip, 1994)

Chronology: We remodeled seven radiocarbon ages reported from analysis of bulk sediment samples (Hope and Tulip, 1994) using Bacon 2.5.0 (Blaauw and Christen, 2011) in R (R Core Team, 2023) (Fig. S28). We ran the model in 101 sections using the Southern Hemisphere calibration curve (SHCal20) (Hogg et al., 2020) and a sediment accumulation rate (acc.mean) of 50 years per cm estimated from linear interpolation between adjacent date samples. We set an assumed age of -40 cal yrs BP (1990 CE) for the core top (0 cm) and set the basal depth for the core at 1000 cm (d.max = 1000).

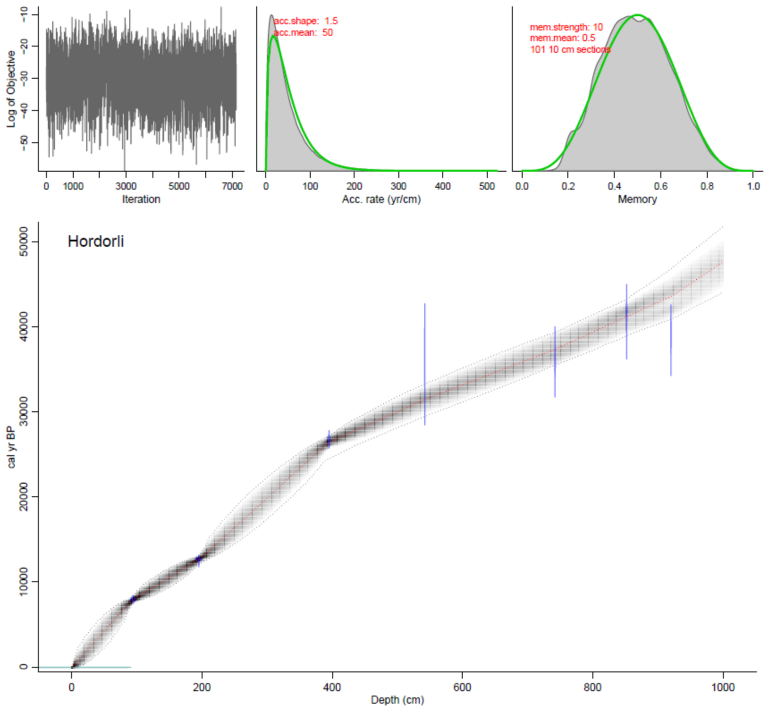

Fig. S28: Age-depth model produced for Lake Hordorli (Hope and Tulip, 1994).

Pollen data preparation: We extracted percentage grass data from the Hope and Tulip (1994) using WebPlotDigitizer (Rohatgi, 2021).

Mixed biogeographic signals

SH1-9014 – Sahul (Northern Australia) and Wallacea (Banda Sea) (van der Kaars et al., 2000)

Chronology for grass pollen: We remodeled nine radiocarbon ages for the core (0 to 185cm) from analysis of planktonic foraminifera (van der Kaars et al., 2000), and nine additional tie points from sediments greater than 297cm determined from $\delta^{18}O$ benthic foraminifera tuning (van der Kaars et al., 2000) and assigned at error of +/-1000, using Bacon 2.5.0 (Blaauw and Christen, 2011) in R (R Core Team, 2023) (Table S3) (Fig. S29). We ran the model in 154 sections using the marine calibration curve Marine20 (Heaton et al., 2020) and a sediment accumulation rate (acc.mean) of 200 years per cm estimated from linear interpolation between adjacent date samples. We set an assumed age of -40 cal yrs BP for the core top (0 cm) and set the basal depth at 764 cm (d.max = 764).

Table S3: 14C and age-tie points used to remodel the chronology of SH1-2014

labID	age	error	depth	cc
surface	-40	10	0	0
0-2	3060	70	1	2
4-6	2640	60	5	2
39-40	13390	110	39	2
75-77	19910	190	76	2
98.5-100	23860	230	99	2
135-136	27890	360	135	2
139-140	29620	390	139	2
156-157	32150	550	156	2
185-186	35580	740	185	2

TP1	51570	1000	298	0
TP2	57600	1000	321	0
TP3	71120	1000	381	0
TP4	79250	1000	431	0
TP5	92230	1000	479	0
TP6	112280	1000	574	0
TP7	125000	1000	600	0
TP8	139020	1000	650	0
TP9	167700	1000	740	0

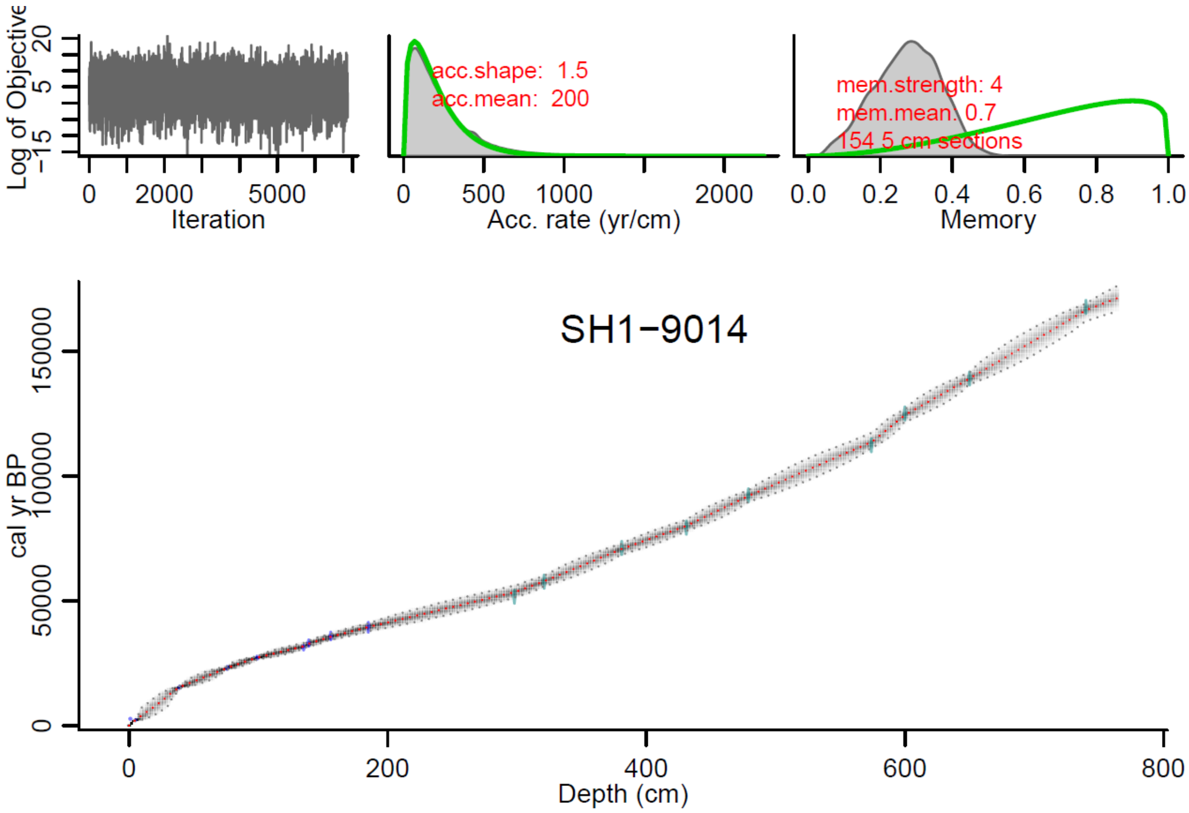

Fig. S29: Age-depth model produced for SH1-9014 (van der Kaars et al., 2000)

Pollen data preparation: We extracted grouped mangrove forest, lowland-submontane forest, montane forest, grassland and woodland forest percentage data from van der Kaars et al. (2000) using WebPlotDigitizer (Rohatgi, 2021) and recalculated them data to reflect percentage of the dryland sum. We extracted grass data, expressed as a percent of the total tree pollen count, from van der Kaars (1998) using WebPlotDigitizer (Rohatgi, 2021) and recalculated them to reflect percentage of the dryland (grass and tree) sum.

G5-6-149P2 – Sahul (West Australia) and Wallacea (Timor Trench) (van der Kaars, 1991)

Chronology: We remodeled four radiocarbon ages for the core, reported from analysis of planktonic foraminifera (van der Kaars, 1991) using Bacon 2.5.0 (Blaauw and Christen, 2011) in R (R Core Team, 2023) (Fig. S30). We ran the model in 142 sections using the marine calibration curve (Marine20) (Heaton et al., 2020) and a sediment accumulation rate (acc.mean) of 100 years per cm estimated from linear interpolation between adjacent date samples. We set an assumed age of -40 cal yrs BP for the core top (0 cm), and set the basal depth for the core at 1412 cm (d.max = 1412).

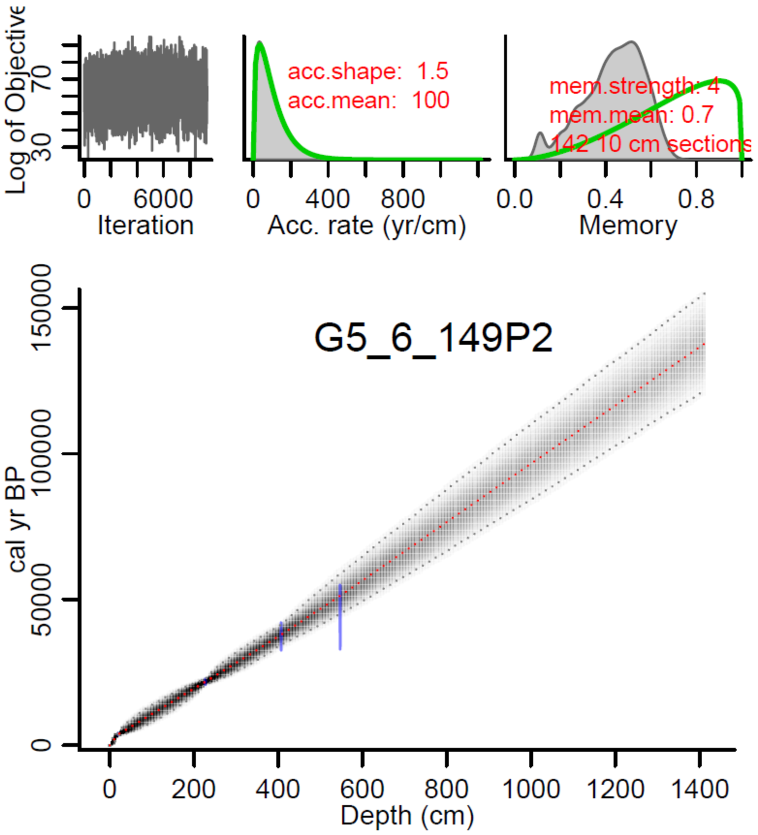

Fig. S30: Age-depth model produced for G5-6-149P2 (van der Kaars, 1991).

Pollen data preparation: We extracted grouped montane forest, mangrove forest, lowland forest, grassland and woodland pollen percentage data from van der Kaars (1991) using WebPlotDigitizer (Rohatgi, 2021), and recalculated them to reflect percentage of the dryland sum by removing the mangrove data. We also removed data from depths greater than 900 cm due to the lack of chronological resolution for the deeper sediments (Fig. S30).

G6-4 – Sahul-Wallacea-Sunda mixed signal (Lombok Ridge) (van der Kaars, 1991)

Chronology: We remodeled five radiocarbon ages reported from analysis of Pteropods (van der Kaars, 1991) using Bacon 2.5.0 (Blaauw and Christen, 2011) in R (R Core Team, 2023) (Fig. S31). We ran the model in 182 sections using the marine calibration curve (Marine20) (Heaton et al., 2020) and a sediment accumulation rate (acc.mean) of 200 years per cm estimated from linear interpolation between adjacent date samples. We set an assumed age of -40 cal yrs BP (1990 CE) was set for the core top, and the basal depth for the core was set at 900 cm (d.max = 900).

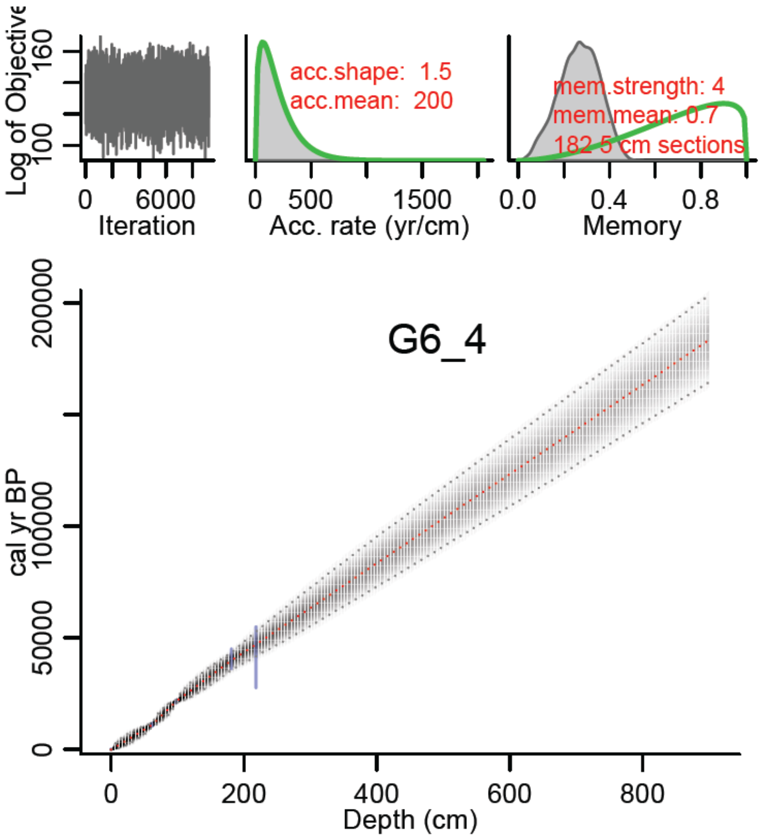

Fig. S31: Age-depth model produced for G6-4 (van der Kaars, 1991)

Pollen data preparation: We extracted grouped montane forest, mangrove forest, lowland forest, grassland and woodland pollen percentage data from van der Kaars (1991) using WebPlotDigitizer (Rohatgi, 2021), and recalculated them to reflect percentage of the dryland sum by removing the mangrove data. We also removed data from depths greater than 400cm due to the lack of chronological resolution for the deeper sediments (Fig. S31).

References

Anshari, G., Peter Kershaw, A., Van Der Kaars, S., Jacobsen, G., 2004. Environmental change and peatland forest dynamics in the Lake Sentarum area, West Kalimantan, Indonesia. Journal of Quaternary Science 19, 637-655.

Bian, Y., Jian, Z., Weng, C., Kuhnt, W., Bolliet, T., Holbourn, A., 2011. A palynological and palaeoclimatological record from the southern Philippines since the Last Glacial Maximum. Chinese Science Bulletin 56, 2359-2365.

Bird, M.I., Boobyer, E.M., Bryant, C., Lewis, H.A., Paz, V., Stephens, W.E., 2007. A long record of environmental change from bat guano deposits in Makangit Cave, Palawan, Philippines. Earth and Environmental Science Transactions of the Royal Society of Edinburgh 98, 59-69.

Birdsell, J., 1977. The Recalibration of a Paradigm for the First Peopling of Greater Australia, in: Allen, J., Golson, J., Jones, R. (Eds.), Sunda and Sahul: Prehistoric Studies in Southeast Asia, Melanesia, and Australia. Academic Press, London, pp. 113-167.

Blaauw, M., Christen, J.A., 2011. Flexible Paleoclimate Age-Depth Models Using an Autoregressive Gamma Process. Bayesian Analysis 6, 457-474.

Dam, R.A.C., Fluin, J., Suparan, P., van der Kaars, S., 2001. Palaeoenvironmental developments in the Lake Tondano area (N. Sulawesi, Indonesia) since 33,000yr B.P. Palaeogeography, Palaeoclimatology, Palaeoecology 171, 147-183.

Dubois, N., Oppo, D.W., Galy, V.V., Mohtadi, M., van der Kaars, S., Tierney, J.E., Rosenthal, Y., Eglinton, T.I., Lückge, A., Linsley, B.K., 2014. Indonesian vegetation response to changes in rainfall seasonality over the past 25,000 years. Nature Geoscience 7, 513-517.

Dubois, N.O., D.W.; Galy, V.; Mohtadi, M.; van der Kaars, S.; Tierney, J.E.; Rosenthal, Y.; Eglinton, T.I., 2014. Indonesian Vegetation and d13C Fatty Acids Data over the Past 25,000 Years. NOAA, Washington D.C.

Flenley, J.R., 1996. Problems of the quaternary on mountains of the Sunda-Sahul region. Quaternary Science Reviews 15, 549-555.

Hamilton, R., Penny, D., Hua, Q., 2019a. A 4700-year record of hydroclimate variability over the Asian monsoon intersection zone inferred from multi-proxy analysis of lake sediments. Global and Planetary Change 174, 92-104.

Hamilton, R., Stevenson, J., Li, B., Bijaksana, S., 2019b. A 16,000-year record of climate, vegetation and fire from Wallacean lowland tropical forests. Quaternary Science Reviews 224.

Hanebuth, T.J.J., Stattegger, K., 2004. Depositional sequences on a late Pleistocene–Holocene tropical siliciclastic shelf (Sunda Shelf, southeast Asia). Journal of Asian Earth Sciences 23, 113-126.

Hanebuth, T.J.J., Stattegger, K., Sidi, F.H., Nummedal, D., Imbert, P., Darman, H., Posamentier, H.W., 2003. The Stratigraphic Evolution of the Sunda Shelf During the Past Fifty Thousand Years, Tropical Deltas of Southeast Asia—Sedimentology, Stratigraphy, and Petroleum Geology. SEPM Society for Sedimentary Geology, p. 0.

Heaton, T.J., Köhler, P., Butzin, M., Bard, E., Reimer, R.W., Austin, W.E.N., Bronk Ramsey, C., Grootes, P.M., Hughen, K.A., Kromer, B., Reimer, P.J., Adkins, J., Burke, A., Cook, M.S., Olsen, J., Skinner, L.C., 2020. Marine20—The Marine Radiocarbon Age Calibration Curve (0–55,000 cal BP). Radiocarbon 62, 779-820.

Hogg, A.G., Heaton, T.J., Hua, Q., Palmer, J.G., Turney, C.S.M., Southon, J., Bayliss, A., Blackwell, P.G., Boswijk, G., Bronk Ramsey, C., Pearson, C., Petchey, F., Reimer, P., Reimer, R., Wacker, L., 2020. SHCal20 Southern Hemisphere Calibration, 0–55,000 Years cal BP. Radiocarbon 62, 759-778.

Hope, G., 2001. Environmental change in the Late Pleistocene and later Holocene at Wanda site, Soroako, South Sulawesi, Indonesia. Palaeogeography, Palaeoclimatology, Palaeoecology 171, 129-145.

Hope, G., Tulip, J., 1994. A long vegetation history from lowland Irian Jaya, Indonesia. Palaeogeography, Palaeoclimatology, Palaeoecology 109, 385-398.

Hunt, C.O., Gilbertson, D.D., Rushworth, G., 2012. A 50,000-year record of late Pleistocene tropical vegetation and human impact in lowland Borneo. Quaternary Science Reviews 37, 61-80.

Kershaw, A.P., van der Kaars, S., Flenley, J.R., 2011. The Quaternary history of Far Eastern rainforests, in: Bush, M., Flenley, J., Gosling, W. (Eds.), Tropical Rainforest Responses to Climatic Change. Springer Berlin Heidelberg, Berlin, Heidelberg, pp. 85-123.

Maloney, B.K., 1980. Pollen analytical evidence for early forest clearance in North Sumatra. Nature 287, 324-326.

Maloney, B.K., McCormac, F.G., 1995. A 30,000-Year Pollen and Radiocarbon Record from Highland Sumatra as Evidence for Climatic Change. Radiocarbon 37, 181-190.

McCarthy, R., Hamdi, R., Erni, Bird, M.I., Wurster, C.M., 2022. Tropical environmental change in North Sumatra at the Last Glacial Maximum: Evidence from the stable isotope composition of cave guano. Palaeogeography, Palaeoclimatology, Palaeoecology 602.

Mohtadi, M., Oppo, D.W., Steinke, S., Stuut, J.-B.W., De Pol-Holz, R., Hebbeln, D., Lückge, A., 2011a. Age model and palaeoclimate records over the past 22,000 years of sediment core GeoB10053-7, Supplement to: Mohtadi, M et al. (2011): Glacial to Holocene swings of the Australian–Indonesian monsoon. Nature Geoscience, 4(8), 540-544, https://doi.org/10.1038/ngeo1209. PANGAEA.

Mohtadi, M., Oppo, D.W., Steinke, S., Stuut, J.-B.W., De Pol-Holz, R., Hebbeln, D., Lückge, A., 2011b. Glacial to Holocene swings of the Australian–Indonesian monsoon. Nature Geoscience 4, 540-544.

Mohtadi, M., Prange, M., Oppo, D.W., De Pol-Holz, R., Merkel, U., Zhang, X., Steinke, S., Lückge, A., 2014. North Atlantic forcing of tropical Indian Ocean climate. Nature 509, 76-80.

Newsome, J., Flenley, J.R., 1988. Late Quaternary Vegetational History of the Central Highlands of Sumatra. II. Palaeopalynology and Vegetational History. Journal of Biogeography 15, 555-578.

Niedermeyer, E.M., Sessions, A.L., Feakins, S.J., Mohtadi, M., 2014a. Hydroclimate of the western Indo-Pacific Warm Pool during the past 24,000 years. Proc Natl Acad Sci U S A 111, 9402-9406.

Niedermeyer, E.M., Sessions, A.L., Feakins, S.J., Mohtadi, M., 2014b. Stable carbon and hydrogen isotope record of n-Alkanoic acids of sediment core SO189/2_144KL, Supplement to: Niedermeyer, EM et al. (2014): Hydroclimate of the western Indo-Pacific Warm Pool during the past 24,000 years. Proceedings of the National Academy of Sciences, 111(26), 9402-9406, https://doi.org/10.1073/pnas.1323585111. PANGAEA.

Penny, D., 2001. A 40,000 year palynological record from north-east Thailand; implications for biogeography and palaeo-environmental reconstruction. Palaeogeography, Palaeoclimatology, Palaeoecology 171, 97-128.

R Core Team, 2023. R: A language and environment for statistical computing. R Foundation for Statistical Computing, Vienna, Austria.

Rohatgi, A., 2021. WebPlotDigitizer 4.5. https://automeris.io/WebPlotDigitizer, Pacifica, California, USA.

Ruan, Y., Mohtadi, M., van der Kaars, S., Dupont, L.M., Hebbeln, D., Schefuß, E., 2018a. N-alkane homologue records (stable hydrogen and carbon isotopes, concentrations) of sediment core GeoB10053-7, In: Ruan, Y et al. (2018): Palynological records and n-alkane records of sediment core GeoB10053-7. PANGAEA, https://doi.org/10.1594/PANGAEA.896146. PANGAEA.

Ruan, Y., Mohtadi, M., van der Kaars, S., Dupont, L.M., Hebbeln, D., Schefuß, E., 2018b. Palynological records and n-alkane records of sediment core GeoB10053-7. PANGAEA.

Ruan, Y., Mohtadi, M., van der Kaars, S., Dupont, L.M., Hebbeln, D., Schefuß, E., 2018c. Palynological records of sediment core GeoB10053-7, In: Ruan, Y et al. (2018): Palynological records and n-alkane records of sediment core GeoB10053-7. PANGAEA, https://doi.org/10.1594/PANGAEA.896146. PANGAEA.

Ruan, Y., Mohtadi, M., van der Kaars, S., Dupont, L.M., Hebbeln, D., Schefuß, E., 2019. Differential hydro-climatic evolution of East Javanese ecosystems over the past 22,000 years. Quaternary Science Reviews 218, 49-60.

Russell, J.M., Vogel, H., Konecky, B.L., Bijaksana, S., Huang, Y., Melles, M., Wattrus, N., Costa, K., King, J.W., 2014. Glacial forcing of central Indonesian hydroclimate since 60,000 y B.P. Proc Natl Acad Sci U S A 111, 5100-5105.

Russell, J.M.V., H.; Konecky, B.L.; Bijaksana, S.; Huang, Y.; Melles, M.; Wattrus, N.; Costa, K.; King, J.W., 2014. Lake Towuti, Indonesia 60KYr Multiproxy Sediment Data. NOAA, Washington D.C.

Spratt, R.M., Lisiecki, L.E., 2016. A Late Pleistocene sea level stack. Climate of the Past 12, 1079-1092.

Stott, L., Poulsen, C., Lund, S., Thunell, R., 2002. Super ENSO and Global Climate Oscillations at Millennial Time Scales. Science 297, 222-226.

Stott, L.D., 2007. Comment on "Anomalous radiocarbon ages for foraminifera shells" by W. Broecker et al.: A correction to the western tropical Pacific MD9821-81 record. Paleoceanography 22.

Stuijts, I.-L.M., 1993. Late Pleistocene and Holocene vegetation of West-Java, Indonesia: Modern quaternary research in Southeast Asia. A.A. Balkema, Rotterdam.

Sun, X., Li, X., Luo, Y., 2002. Vegetation and climate on the sunda shelf of the South China Sea during the last Glactiation--Pollen results from station 17962. Acta Botanica Sinica 44, 746-752.

Sun, X., Li, X., Luo, Y., Chen, X., 2000. The vegetation and climate at the last glaciation on the emerged continental shelf of the South China Sea. Palaeogeography, Palaeoclimatology, Palaeoecology 160, 301-316.

Thilakanayaka, V., Chuanxiu, L., Xiang, R., Devendra, D., Dasanayaka, S.A.H.K., Jiang, W., Rahman, A., Kumar, S., Ariful, G.M., 2019. Sediment Provenance of the Nansha Trough Since 40 ka B.P. in the South China Sea: Evidence From δ13Corg, TOC and Pollen Composition. Frontiers in Earth Science 7.

van der Kaars, S., 1998. Marine and Terrestrial Pollen Records of the Last Glacial Cycle from the Indonesian Region: Bandung Basin and Banda Sea. Palaeoclimates 3, 209–219.
van der Kaars, S., Bassinot, F., De Deckker, P., Guichard, F., 2010. Changes in monsoon and ocean circulation and the vegetation cover of southwest Sumatra through the last 83,000years: The record from marine core BAR94-42. Palaeogeography, Palaeoclimatology, Palaeoecology 296, 52-78.
van der Kaars, S., Dam, R., 1997. Vegetation and climate change in West-Java, Indonesia during the last 135,000 years. Quaternary International 37, 67-71.
van der Kaars, S., Penny, D., Tibby, J., Fluin, J., Dam, R.A.C., Suparan, P., 2001. Late Quaternary palaeoecology, palynology and palaeolimnology of a tropical lowland swamp: Rawa Danau, West-Java, Indonesia. Palaeogeography, Palaeoclimatology, Palaeoecology 171, 185-212.
van der Kaars, S., Wang, X., Kershaw, P., Guichard, F., Setiabudi, D.A., 2000. A Late Quaternary palaeoecological record from the Banda Sea, Indonesia: patterns of vegetation, climate and biomass burning in Indonesia and northern Australia. Palaeogeography, Palaeoclimatology, Palaeoecology 155, 135-153.
van der Kaars, W.A., 1991. Palynology of eastern Indonesian marine piston-cores: a Late Quaternary vegetational and climatic record for Australasia. Palaeogeography, Palaeoclimatology, Palaeoecology 85, 239-302.
van der Kaars, W.A., Dam, M.A.C., 1995. A 135,000-year record of vegetational and climatic change from the Bandung area, West-Java, Indonesia. Palaeogeography, Palaeoclimatology, Palaeoecology 117, 55-72.
Walker, D., Flenley, J.R., 1979. Late Quaternary vegetational history of the Enga province of Upland Papua New Guinea. Phil. Trans. R. Soc. Lond. B 286, 265-344.
Wang, X., Sun, X., Wang, P., Stattegger, K., 2009. Vegetation on the Sunda Shelf, South China Sea, during the Last Glacial Maximum. Palaeogeography, Palaeoclimatology, Palaeoecology 278, 88-97.
Wicaksono, S.A., Russell, J.M., Bijaksana, S., 2015. Compound-specific carbon isotope records of vegetation and hydrologic change in central Sulawesi, Indonesia, since 53,000 yr BP. Palaeogeography, Palaeoclimatology, Palaeoecology 430, 47-56.
Wicaksono, S.A., Russell, J.M., Holbourn, A., Kuhnt, W., 2017. Hydrological and vegetation shifts in the Wallacean region of central Indonesia since the Last Glacial Maximum. Quaternary Science Reviews 157, 152-163.
Wurster, C.M., Bird, M.I., Bull, I.D., Creed, F., Bryant, C., Dungait, J.A.J., Paz, V., 2010. Forest contraction in north equatorial Southeast Asia during the Last Glacial Period. Proceedings of the National Academy of Sciences 107, 15508-15511.
Wurster, C.M., Rifai, H., Zhou, B., Haig, J., Bird, M.I., 2019. Savanna in equatorial Borneo during the late Pleistocene. Scientific Reports 9.
Xiang, R., Chen, M., Li, Q., Liu, J., Zhang, L., Lu, J., 2009. Planktonic foraminiferal records of East Asia monsoon changes in the southern South China Sea during the last 40,000years. Marine Micropaleontology 73, 1-13.
Yang, Z., Lei, Y., Rosenthal, Y., Li, T., Jian, Z., 2021. Late Pleistocene climate induced changes in paleo-vegetation in Borneo: Possible implications to human divergence. Quaternary Science Reviews 267.
